# Improving spatial prediction of *Schistosoma haematobium* prevalence in southern Ghana through new remote sensors and local water access profiles

**DOI:** 10.1371/journal.pntd.0006517

**Published:** 2018-06-04

**Authors:** Alexandra V. Kulinkina, Yvonne Walz, Magaly Koch, Nana-Kwadwo Biritwum, Jürg Utzinger, Elena N. Naumova

**Affiliations:** 1 Department of Civil and Environmental Engineering, Tufts University, Medford, Massachusetts, United States of America; 2 Institute for Environment and Human Security, United Nations University, Bonn, Germany; 3 Center for Remote Sensing, Boston University, Boston, Massachusetts, United States of America; 4 Neglected Tropical Diseases Program, Ghana Health Service, Accra, Ghana; 5 Swiss Tropical and Public Health Institute, Basel, Switzerland; 6 University of Basel, Basel, Switzerland; 7 Friedman School of Nutrition Science and Policy, Tufts University, Boston, Massachusetts, United States of America; Case Western Reserve University School of Medicine, UNITED STATES

## Abstract

**Background:**

Schistosomiasis is a water-related neglected tropical disease. In many endemic low- and middle-income countries, insufficient surveillance and reporting lead to poor characterization of the demographic and geographic distribution of schistosomiasis cases. Hence, modeling is relied upon to predict areas of high transmission and to inform control strategies. We hypothesized that utilizing remotely sensed (RS) environmental data in combination with water, sanitation, and hygiene (WASH) variables could improve on the current predictive modeling approaches.

**Methodology:**

*Schistosoma haematobium* prevalence data, collected from 73 rural Ghanaian schools, were used in a random forest model to investigate the predictive capacity of 15 environmental variables derived from RS data (Landsat 8, Sentinel-2, and Global Digital Elevation Model) with fine spatial resolution (10–30 m). Five methods of variable extraction were tested to determine the spatial linkage between school-based prevalence and the environmental conditions of potential transmission sites, including applying the models to known human water contact locations. Lastly, measures of local water access and groundwater quality were incorporated into RS-based models to assess the relative importance of environmental and WASH variables.

**Principal findings:**

Predictive models based on environmental characterization of specific locations where people contact surface water bodies offered some improvement as compared to the traditional approach based on environmental characterization of locations where prevalence is measured. A water index (MNDWI) and topographic variables (elevation and slope) were important environmental risk factors, while overall, groundwater iron concentration predominated in the combined model that included WASH variables.

**Conclusions/Significance:**

The study helps to understand localized drivers of schistosomiasis transmission. Specifically, unsatisfactory water quality in boreholes perpetuates reliance on surface water bodies, indirectly increasing schistosomiasis risk and resulting in rapid reinfection (up to 40% prevalence six months following preventive chemotherapy). Considering WASH-related risk factors in schistosomiasis prediction can help shift the focus of control strategies from treating symptoms to reducing exposure.

## Author summary

Schistosomiasis is a water-related neglected tropical disease that disproportionately affects school-aged children in poor communities of low- and middle-income countries. Schistosomiasis transmission risk is affected by environmental, socioeconomic, and behavioral factors, including water, sanitation, and hygiene (WASH) conditions. We used fine spatial resolution (10–30 m) remotely sensed data, in combination with measures of local water access and groundwater quality, to predict schistosomiasis risk in 73 rural Ghanaian communities. We found that applying environmental models to specific locations where people contact surface water bodies (i.e., potential transmission locations), rather than to locations where prevalence is measured, improved model performance. A remotely sensed water index and topographic variables (elevation and slope) were important environmental risk factors, while overall, groundwater iron concentration predominated. In the study area, unsatisfactory water quality in boreholes perpetuates reliance of surface water bodies, indirectly increasing schistosomiasis risk and resulting in rapid reinfection (up to 40% prevalence six months following deworming). Considering WASH-related risk factors in schistosomiasis prediction can help shift the focus of control strategies from treating symptoms to reducing exposure.

## Introduction

Schistosomiasis is an important parasitic disease that affects more than 250 million people [1]. Expressed in years lived with disability (YLDs), the impact of schistosomiasis is comparable to that of malaria (2.9 versus 3.2 million YLDs) [2]. Schistosomiasis is a disease of poverty, with 97% of all infections and 85% of the global at-risk population concentrated in Africa [3]. Ghana has an estimated country-wide prevalence of 23.3%, with focal, or localized, prevalence levels >50% [4].

Schistosomiasis is caused by infection with the trematode parasite of the genus *Schistosoma* [5]. Of the three species that commonly infect humans (*S*. *haematobium*, *S*. *mansoni*, and *S*. *japonicum*), the former two are prevalent in Africa [6]. *S*. *haematobium* is the predominant species in Ghana [4] and is the focus of the present study. Schistosomiasis has a complex life cycle that involves the parasite, intermediate host snails, and definitive human host (and sometimes animal reservoir hosts). Transmission occurs in fresh surface water bodies that are contaminated with human waste, provide favorable ecologic conditions for intermediate host snails (*Bulinus* species for *S*. *haematobium*), and sustain human water contact [6]. Human transmission occurs when parasite larvae (cercariae) penetrate intact skin during water-based activities and has historically been most common in rural areas with natural slow flowing streams, ponds, and lakes [3,6].

To develop and implement effective control strategies against schistosomiasis, accurate data on the geographic and demographic distribution of infections are necessary. Surveillance in endemic low- and middle-income countries is inhibited by limited health infrastructure and cases evading clinical detection due to lower parasite burden and lessened symptoms that result from preventive chemotherapy with the anthelmintic drug praziquantel. Passive health facility-based surveillance and reporting systems are known to severely underestimate the number of infections [7,8]. For example, a total of ~25,000 schistosomiasis cases were reported into the Ghanaian District Health Information Management System (DHIMS) in 2010 (data received from GHS, 2016). If only ~5 million children ≤15 years of age residing in rural areas (i.e., high-risk population) [9] are considered at the estimated 23.3% infection rate [4], ~1.15 million cases would be expected. The reported cases represent only 2.2% of this expected number. Some correction for underreporting can be accomplished by predictive modeling, aiming to complement data from surveillance systems and field-based prevalence surveys.

Many schistosomiasis predictive modeling studies have been published and reviewed [10,11]. Most studies utilized remote sensing (RS) and geographic information system (GIS) approaches at large spatial extents (i.e., national, regional or continental) [12–14], with fewer applications of these methods to sub-national mapping [15–17]. Because snail populations, cercarial densities, human water contact patterns, and subsequent schistosomiasis infections exhibit strong spatial heterogeneity [10,18,19], further investigation of localized transmission drivers at smaller spatial extents is needed [10,11]. Furthermore, most studies included relatively few RS environmental predictors, mainly normalized difference vegetation index (NDVI), land surface temperature (LST), and elevation, whereas many other vegetation- and moisture-related indices and topographic variables are available and should be considered [11,20,21].

Another important limitation is that most studies utilized point-prevalence data of human infections (outcome) typically measured at schools, whereas RS-based environmental data (predictors) pertain to water bodies that serve as snail habitats and potential transmission locations. Most models do not account for this spatial mismatch between exposure and outcome measures [11]. A recent study used a more ecologically relevant approach, in which RS variables were extracted from geographically delineated water bodies within a buffer radius around the point-prevalence location [22]. An even more promising approach would be to apply the models to the specific locations along water bodies where human water contacts occur.

Further complicating the modeling approach at small spatial extents are socioeconomic and behavioral factors, including water, sanitation, and hygiene (WASH) conditions, known to affect individual schistosomiasis risk [23–25]. These factors may have an even greater bearing on the focal nature of disease distribution than the environment [26,27], and should be considered as predictors. While the inclusion of socioeconomic status and metrics of clean water and sanitation access have been advocated [10,11], to our knowledge, WASH variables have not yet been explicitly incorporated into spatial schistosomiasis predictive models.

The goal of the present study was to build upon existing predictive modeling approaches using *S*. *haematobium* prevalence data from 73 rural communities in the Eastern region of Ghana. We utilized fine resolution RS data (Landsat 8 and Sentinel-2), expanded the number of predictors (15 environmental and four WASH-related variables), and explored alternatives for addressing the spatial mismatch between exposure and outcome measures. In this study, primary innovations include the use of a new RS data source (Sentinel-2), incorporation of field-mapped surface water contact sites into the RS-based environmental modeling approach, and exploration of WASH variables as additional schistosomiasis risk factors.

## Methods

### Ethics statement

The study was approved by the Institutional Review Board (IRB) at Tufts University in Boston, United States of America (protocol #11688) and Noguchi Memorial Institute for Medical Research in Accra, Ghana (protocol #1133). Letters of approval were obtained from national and regional offices of Ghana Health Service (GHS) and Ghana Education Service (GES). Written informed consent was obtained from the acting head teacher of each school that participated in the schistosomiasis prevalence survey. Verbal assent was sought from the participating children, an accepted ethical and practical approach used in similar low-risk studies [28].

### Study area

**Fig 1:**
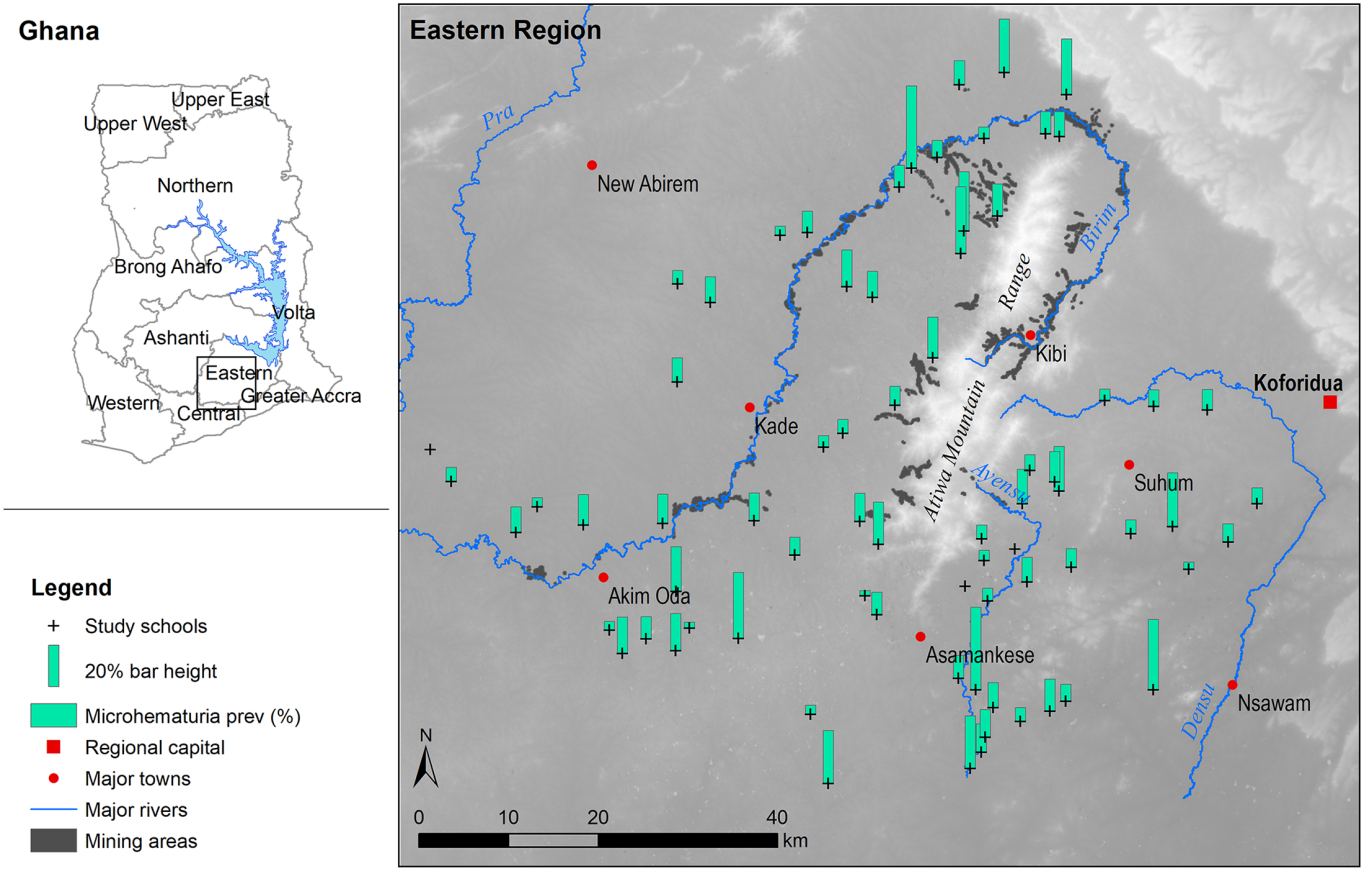
Map of the study area and spatial distribution of microhematuria (typical symptom of *S*. *haematobium* in school-aged children) prevalence created using the following data sources: Major rivers and town locations were obtained from CERSGIS, Accra, Ghana; hillshade relief surface was created from elevation data obtained from ASTER Global Digital Elevation Model (v2); mining locations were digitized from Sentinel-2 satellite imagery; microhematuria prevalence data were collected by A. Kulinkina.

The study was conducted in the tropical Eastern region (Fig 1), characterized by major and minor peak rainfall periods in June and October, respectively, with dry season lasting from November to February. Four major perennial rivers (Pra, Birim, Ayensu, and Densu) drain the region, with an abundance of smaller streams and ponds. Most of these water bodies are used extensively for domestic and recreational purposes (e.g., fetching, washing, swimming, and fishing). The Pra and Birim rivers, and some of their tributaries, however, are heavily polluted by alluvial gold mining and are no longer used due to high turbidity and presence of toxic compounds [29]. The region is relatively flat with some hilly areas and low mountains (Atiwa Mountain Range) reaching an elevation of approximately 750 m above sea level. The study area, spanning 10 administrative districts, was purposely selected outside of a 20-km buffer radius of Lake Volta [29]. Communities situated on its shores are historically known to be endemic for schistosomiasis [30]. However, little information is available about pockets of high transmission along minor rivers and streams that are not easily detected with RS technologies.

### Community as a unit of analysis

**Fig 2:**
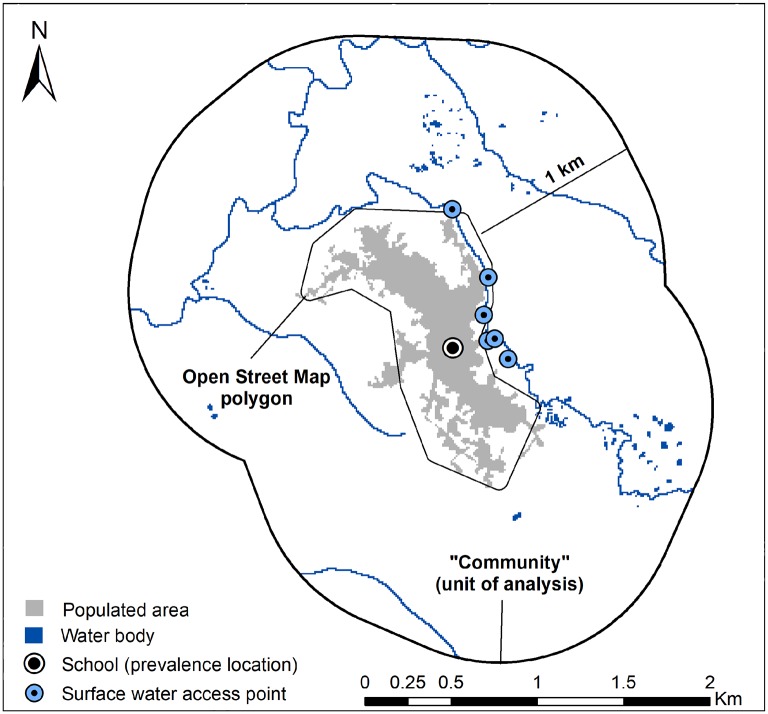
Spatial definitions associated with the analysis conducted at the “community” level.

Prior modeling studies mainly used point-prevalence as outcome data. Prevalence of *S*. *haematobium* eggs in urine samples (or hematuria as a proxy of infection) is typically measured at schools, while transmission may occur within some distance of this point-prevalence location. With extensive local knowledge from prior community-based studies [29,31–33], the present analysis was conducted at the “community” level. The spatial boundaries of communities were defined by Open Street Map (OSM) polygons (Fig 2) abstracted using QGIS software (version 2.12.3), an approach validated in a case study [29]. Subsequently, a buffer radius of 1 km was applied to each polygon. The buffer distance was chosen because nearly all known contact with water bodies occurred within 1 km of community boundaries. Throughout the manuscript, the term “community” refers to the OSM polygon + 1 km buffer area (Fig 2) and is used as a unit of analysis, also referred to as grain or support [21,34].

### Data sources

Data for this study were obtained primarily from satellite RS sources and field studies, with some additional geographic features digitized from satellite imagery. Surface reflectance, thermal, and elevation data were obtained from RS sources. From these, vegetation and water indices, LST, and topographic variables were derived. WASH variables were obtained from field data available from past studies, namely global positioning system (GPS) coordinates of public water sources [29] and data about groundwater quality [32]. The outcome variable, *S*. *haematobium* prevalence (%) was measured in one school in each of the 73 study communities. Measures of improved and unimproved [35] water access and groundwater quality (WASH variables) were combined with RS-based variables to predict schistosomiasis prevalence across the study area. Data processing and analysis steps are described below and outlined in S1 and S2 Figs in Supporting Information.

#### Remotely sensed data

Surface reflectance data were obtained from two RS data sources: Landsat 8 Operational Land Imager (OLI) and Sentinel-2. Landsat 8 data were obtained from USGS Earth Explorer (http://earthexplorer.usgs.gov/) and included two cloud-free scenes that were mosaicked to cover the extent of the study area (Table 1). OLI data (bands 2–6 in Table 1) were downloaded as raw digital number (DN) values with a spatial resolution of 30 m and radiometrically and atmospherically corrected to obtain surface reflectance. This two-step procedure consisted of converting DN values to top-of-atmosphere (TOA) radiance, followed by an atmospheric correction using the Fast Line-of-sight Atmospheric Analysis of Hypercubes (FLAASH) module in ENVI 5.4 (Exelis Visual Information Solutions, Boulder, United States of America). Thermal data (bands 10 and 11 in Table 1) were downloaded from Landsat 8 Thermal InfraRed Sensor (TIRS) as level 1 (L1B) products with a spatial resolution of 100 m.

**Table 1:** Summary of surface reflectance data used in the study.

	Landsat 8	Sentinel-2
Scenes (acquisition dates)	Path 193 Row 56 (December 22, 2015) Path 194 Row 56 (December 29, 2015)	T30NXM (December 24, 2015) T30NXN (December 24, 2015) T30NYM (December 24, 2015) T30NYN (December 24, 2015)
Bands (wavelengths)	Blue–B2 (0.450–0.515 μm) Green–B3 (0.525–0.600 μm) Red–B4 (0.630–0.680 μm) Near infrared–B5 (0.845–0.885 μm) Short wavelength infrared–B6 (1.560–1.660 μm) Long wavelength infrared–B10 (10.30–11.30 μm) Long wavelength infrared–B11 (11.50–12.50 μm)	Blue–B2 (0.490 μm) Green–B3 (0.560 μm) Red–B4 (0.665 μm) Near infrared–B8 (0.842 μm) Short wavelength infrared–B11 (1.610 μm)

Sentinel-2 surface reflectance data (bands 2, 3, 4, 8, and 11 in Table 1) were obtained from Copernicus Data Hub (https://scihub.copernicus.eu/). Four cloud-free scenes were mosaicked to cover the extent of the study area (Table 1). TOA radiance (level 1C) products were downloaded with a spatial resolution of 10 or 20 m and converted to level 2A surface reflectance by applying the atmospheric correction using the Sen2Cor processor in open-source Sentinel Application Platform (SNAP) software (version 5.0).

ASTER Global Digital Elevation Model (GDEM v2) data were obtained from USGS Global Data Explorer (gdex.cr.usgs.gov) with a spatial resolution of 30 m. A moving window (3x3) majority filter was applied to the elevation data to eliminate image artefacts [36,37] using the Spatial Analyst extension in ArcGIS 10.2.2.

Settlement data were obtained from the German Aerospace Center (http://www.dlr.de) as a new Global Urban Footprint (GUF) product. GUF is a binary raster data product of populated and unpopulated pixels produced from 2011–2012 TerraSAR-X and TanDEM-X radar images [38]. GUF was chosen as a source of settlement data due to its 0.4 arcsec geometric resolution, or 12 m spatial resolution, which most closely matched the resolution of the other spatial data used in the study.

#### Field data

A cross-sectional *S*. *haematobium* prevalence survey was conducted in May and June 2016 in the largest primary school in each of the 73 study communities (population range 500–5,000). The most recent round of national school-based preventive chemotherapy had been conducted in January 2016 (six months prior to the survey); all study schools had participated, with an average treatment coverage of 78% (data provided by GHS, 2016). All children in grades 3 and 4 (age range 8–13 years) who expressed verbal assent were enrolled into the study. Upon detailed demonstrations of the specimen collection procedure, children were invited to provide a urine sample between 10:00 and 14:00 hours that was tested for microhematuria using a semi-quantitative reagent strip on-site. Samples with any blood presence, including “trace”, were categorized as positive readings [28]. Infected children were offered praziquantel according to their weight by a local nurse or community health worker in a private location. No identifying information about study subjects was recorded besides school/community name, sex, and grade.

A total of 5,220 children (2,802 boys and 2,418 girls) were registered in grades 3 and 4 in the 73 study schools. Of these, 3,746 children (72%) were present on the day of screening. Attendance in some of the schools was as low as 46%. A total of 3,628 children (97%) were enrolled into the study, and 3,612 (>99%) provided urine samples for analysis. Prevalence of microhematuria in the study population was 14%; school-level prevalence values ranged between 0 and 40% (Fig 1; S1 Table, Supporting Information).

### Data processing

Six environmental indices were calculated from Landsat 8 (OLI) and Sentinel-2 surface reflectance data (Table 2) in R software (version 3.3.1). In the enhanced vegetation index (EVI) equation, *L* value adjusts for canopy background and *C* values are coefficients for atmospheric resistance. These enhancements allow for index calculation as a ratio between the red and the near infrared (*nir*) band values, while reducing the background and atmospheric noise and saturation [39]. The values of *C*_*1*_ = 6, *C*_*2*_ = 7.5, and *L* = 1 were obtained from the Landsat 8 product guide [40]. In the soil adjusted vegetation index (SAVI) equation, *L* is the soil calibration factor that minimizes soil background conditions that affect partial canopy spectra. The *L* value of 0.5 minimizes soil brightness variation and eliminates the need for additional calibration for different soils [41]. Landsat 8 (TIRS) thermal data were processed using ATCOR [42] with a standard emissivity of 0.985 to detect water surface temperature, and converted from Kelvin (K) to degrees Celsius (°C) to represent LST.

**Table 2:** Six environmental indices computed with Landsat 8 (OLI) and Sentinel-2 data.

Index	Equation	Reference
Normalized difference vegetation index (NDVI)	$\frac{\left(nir-red\right)}{\left(nir+red\right)}$	[43]
Enhanced vegetation index (EVI)	$\frac{\left(nir-red\right)}{\left(nir+C_{1}*red-C_{2}*blue+L\right)}$	[39]
Soil adjusted vegetation index (SAVI)	$\frac{\left(nir-red\right)}{\left(nir+red+L\right)}(1+L)$	[41]
Modified soil adjusted vegetation index (MSAVI)	$\frac{[2*nir+1-\sqrt{{\left(2*nir+1\right)}^{2}-8(nir-red)]}}{2}$	[44]
Normalized difference water index (NDWI)	$\frac{\left(green-nir\right)}{\left(green+nir\right)}$	[45]
Modified normalized difference water index (MNDWI)	$\frac{\left(green-swir\right)}{\left(green+swir\right)}$	[46]

Elevation data were used to derive stream order and slope. Topographic drainage lines were delineated from the digital elevation model (DEM) based on the potential flow direction from higher to lower elevation and accumulation of surface runoff according to topographic conditions using Arc Hydro Tools in ArcGIS (version 10.2.2). The resulting stream network was ordered according to Strahler [47]. Slope of the terrain was derived from the DEM as a proxy indicator for potential flow velocity of surface runoff with inclination calculated in degrees.

GPS coordinates of public water sources (standpipes (SPs), boreholes (BHs), protected and unprotected hand-dug wells (HDWs), and surface water access points (SWAPs)) were available from a prior study [29]. SPs, BHs, and protected HDWs that were functional at the time of the study constituted functional improved water sources (FIWS) that are not capable of transmitting schistosomiasis. SWAPs constituted unimproved water sources that are capable of transmitting schistosomiasis. Two categorical raster layers were derived from the GPS data using a buffer analysis conducted in ArcGIS 10.2.2, which represented improved water access (within 100–500 m of FIWS) and surface water access (within 100–500 m of SWAP), to test the hypothesis that locations closer to FIWSs have a lower risk of schistosomiasis transmission and locations closer to SWAPs have higher risk of schistosomiasis transmission [29].

Two additional raster layers of interpolated groundwater iron and total dissolved solids (TDS) concentrations (mg/l) were also obtained from a prior study [32]. Groundwater quality variables were included because prior studies [29,32,33] suggested that elevated iron and TDS concentrations in BHs may increase reliance on contaminated surface water bodies, thereby potentially serving as indirect risk factors for schistosomiasis transmission.

Lastly, *S*. *haematobium* prevalence (% positive samples) was calculated from survey data. Prevalence was determined separately for boys and girls in each grade and then adjusted to a gender- and grade- balanced population using direct standardization [48]. Standardized school-level point-prevalence values (S1 Table, Supporting Information) were taken to represent community-level prevalence based on the following validated [49] assumptions: (i) microhematuria prevalence measured by reagent strip is a reasonable proxy of *S*. *haematobium* prevalence in a presumably lightly infected population due to recent preventive chemotherapy; (ii) 3^rd^ and 4^th^ grade school children are a representative study population; and (iii) where a child lives and attends school are not spatially dependent, inferring that prevalence value at one school is representative of community-level prevalence.

### Variable extraction and aggregation

**Fig 3:**
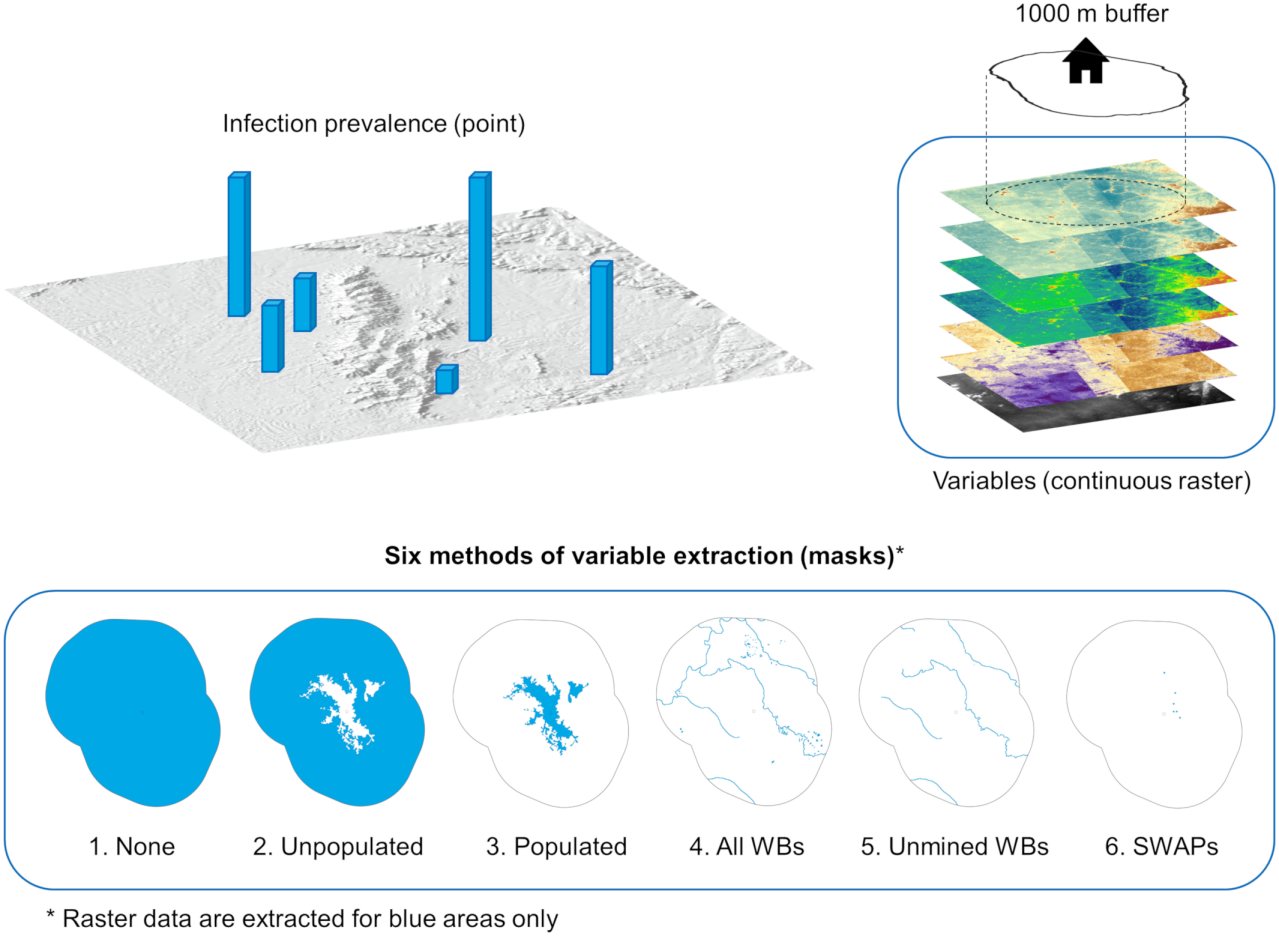
Modeling approach explaining raster data extraction methods to be matched to each point-prevalence location.

A total of 15 environmental and four WASH predictor variables (Table 3; S3–S21 Figs, Supporting Information) were derived and resampled to a matching spatial resolution of 10 m. While *S*. *haematobium* infection prevalence was represented by point data, predictors were represented by continuous raster data (Fig 3). Therefore, extraction and aggregation of the raster data within the “community” polygons were necessary.

**Table 3:** Environmental (top) and WASH (bottom) predictor variables.

Data source	Variable	Scale [range]	Resolution [m]	Mask	Aggregation
OLI/Sentinel	Blue band	Continuous [0–1]	30 / 10	1, 2, 4, 5, 6	Median
OLI/Sentinel	Green band	Continuous [0–1]	30 / 10	1, 2, 4, 5, 6	Median
OLI/Sentinel	Red band	Continuous [0–1]	30 / 10	1, 2, 4, 5, 6	Median
OLI/Sentinel	nir band	Continuous [0–1]	30 / 10	1, 2, 4, 5, 6	Median
OLI/Sentinel	swir band	Continuous [0–1]	30 / 20	1, 2, 4, 5, 6	Median
OLI/Sentinel	NDVI	Continuous [-1-1]	30 / 10	1, 2, 4, 5, 6	Median
OLI/Sentinel	EVI	Continuous [-1-1]	30 / 10	1, 2, 4, 5, 6	Median
OLI/Sentinel	SAVI	Continuous [-1-1]	30 / 10	1, 2, 4, 5, 6	Median
OLI/Sentinel	MSAVI	Continuous [-1-1]	30 / 10	1, 2, 4, 5, 6	Median
OLI/Sentinel	NDWI	Continuous [-1-1]	30 / 10	1, 2, 4, 5, 6	Median
OLI/Sentinel	MNDWI	Continuous [-1-1]	30 / 10	1, 2, 4, 5, 6	Median
TIRS	LST [°C]	Continuous [13–47]	100	1, 2, 4, 5, 6	Median
DEM	Elevation [m]	Continuous [1–870]	30	1, 2, 4, 5, 6	Median
DEM	Slope [°]	Continuous [0–85]	30	1, 2, 4, 5, 6	Median
DEM	Stream order	Categorical [0–5]	30	1	Max
Field data	FIWS access	Categorical [0–5]	--	3	Mode
Field data	SWAP access	Categorical [0–5]	--	3	Mode
Field data	Iron [mg/l]	Continuous [0.1–0.7]	--	1	Median
Field data	TDS [mg/l]	Continuous [83–616]	--	1	Median

A total of six methods of variable extraction (masks) were used (Fig 3): none {1}–all pixels within the “community” polygon were extracted; unpopulated {2}–data were extracted only for unpopulated pixels as defined by the GUF data; populated {3}–data were extracted only for populated pixels as defined by the GUF data; all water bodies {4}–mask was derived by combining the topographic drainage lines from the DEM, supplemented with ponds, lakes, and gold mining pits that were digitized from satellite imagery; unmined water bodies {5}–mask was derived by removing water bodies that are known to be affected by mining from “all water bodies”; SWAPs {6}–defined as the single pixel GPS points of known surface water contact sites.

To understand the spatial linkage between school-based prevalence and the environmental conditions, almost all environmental variables were extracted using masks {1, 2, 4, 5, and 6} (Table 3), listed in the order of increasing ecologic relevance. For example, the most ecologically relevant method is to match school-based schistosomiasis prevalence with environmental variables extracted from points within the “community” where known contact with water bodies occurs (m6). Method 3 (populated areas) was not relevant for environmental variable extraction because these locations are not representative of schistosomiasis transmission. Conversely, measures of safe (FIWS) and unsafe (SWAP) water access apply only to populated areas; hence only method 3 was used to extract these two WASH variables. Unmasked data (m1) were used to extract stream order, iron, and TDS concentrations (Table 3). For aggregation of environmental variables, primarily the median pixel values were used, except for stream order, where maximum value was used. For aggregation of WASH variables, either median (iron and TDS) or mode (FIWS and SWAP access) were used (Table 3).

### Data analysis

Exploratory analyses included variable summaries and correlations, followed by random forest models. The random forest approach was chosen because it can deal with continuous outcome data, multicollinear predictor variables, and low numbers of training samples, it is the recommended machine learning method for generating predictions [50], and it has been successfully applied in similar studies [22].

Five non-parametric random forest models were conducted with 15 environmental predictor variables (Table 3) to determine which of the five masks presented the best method of variable extraction. Two versions of the analyses were conducted in parallel (with Landsat 8 and Sentinel-2 surface reflectance values and environmental indices) to test consistency of predictive performance of RS data obtained from these two satellites with similar acquisition dates. Explanatory power of random forest models was compared using root-mean-square error (RMSE) and R^2^ values [51], and relative importance of predictor variables was assessed using the increasing node purity (“IncNodePurity”) metric [52,53].

All models were applied back to the raster stack of predictor variables to derive continuous predicted *S*. *haematobium* prevalence surfaces. Although predicted values were available for all pixels, the same masks used to extract the explanatory variables were applied to the respective predicted prevalence surfaces. After applying the masks, the median predicted values within each “community” were plotted against observed prevalence values. The quality of prediction was assessed using Spearman’s rank correlation between model predicted and observed values, and their fit was compared to the line of equality. Lastly, environmental data extracted using the best performing mask were combined with the WASH variables in a final model to assess the relative importance of the two groups of variables.

## Results

### Comparison of five environmental variable extraction methods

As an exploratory analysis, Spearman’s rank correlations were computed between pairs of environmental indices (S2 Table, Supporting Information). The correlation values were consistent across extraction masks and across RS data sources. As expected, correlations among the vegetation indices derived using both Landsat 8 and Sentinel-2 data were generally very high (0.90–0.99). Lower correlation values were observed between the two water indices NDWI and MNDWI (~0.70). Consequently, negative correlation values between NDWI and the vegetation indices were much higher than those between MNDWI and the vegetation indices (0.91 versus 0.50).

**Fig 4:**
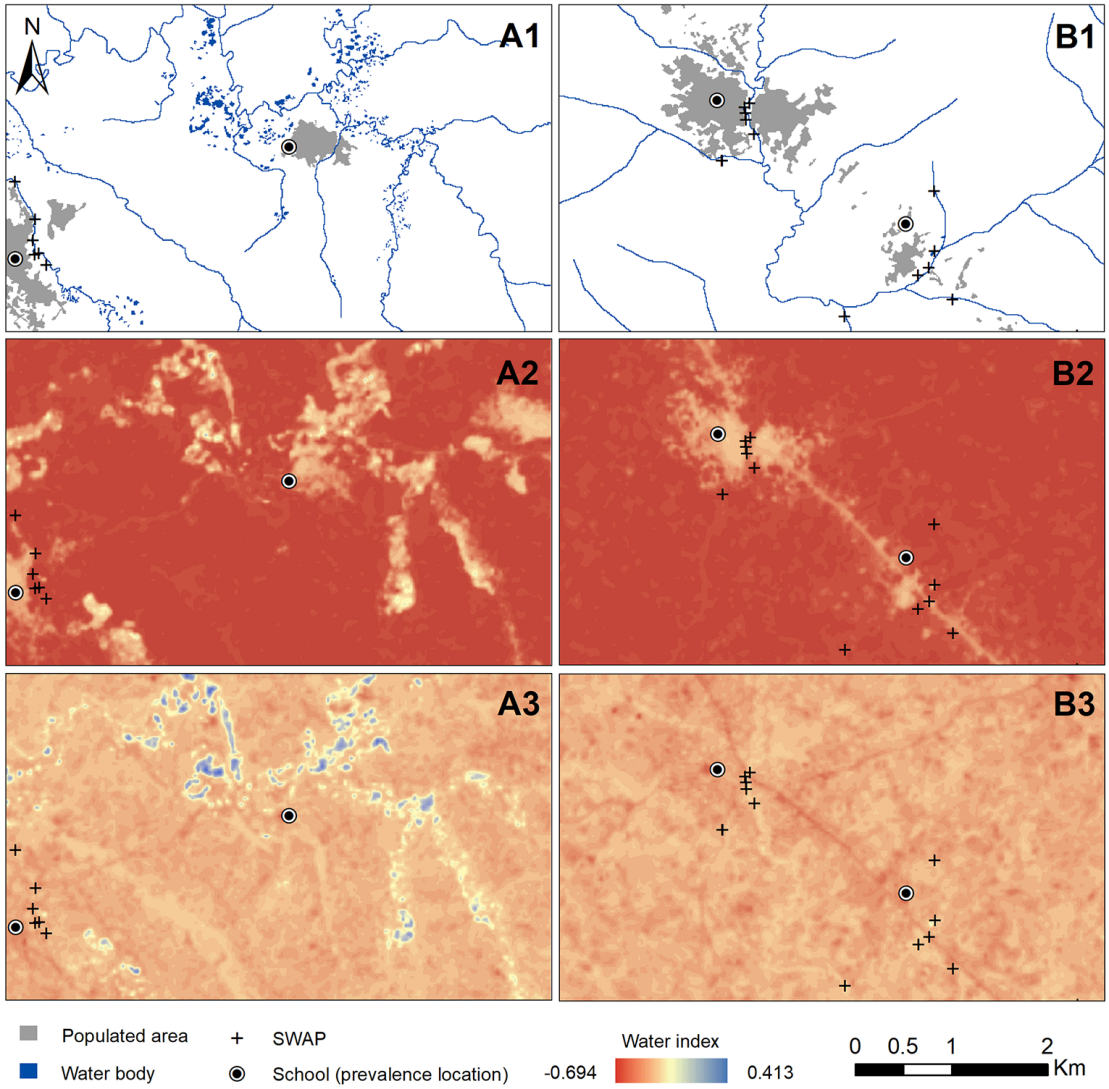
Schematic images of study communities showing settlements and water bodies (A1 and B1), NDWI values (A2 and B2), and MNDWI values (A3 and B3) generated using Landsat 8 data.

To explore the potential reason for this, NDWI and MNDWI were visually compared against a map (Fig 4). In the first row (A1 and B1), schematic maps of study communities are shown with populated areas indicated in gray and water bodies, comprised of rivers/streams and dug mining pits, indicated in blue. It appears that the NDWI computed with Landsat 8 data (A2 and B2) results in false detection of water bodies (i.e., misclassification of developed surfaces such as settlements and roads), essentially serving as an inverse of a vegetation index, which explains the strong negative correlation with vegetation indices. On the other hand, the MNDWI (A3 and B3) more precisely detects water bodies, particularly mining pits. Same conclusions apply to NDWI and MNDWI values derived from Sentinel-2 data (S13 and S14 Figs, Supporting Information). Neither index performed adequately at detecting the SWAPs, shown as + symbols in Fig 4.

Random forest models were first run for each extraction method using environmental variables only (Table 4). Two versions of the environmental models were run in parallel with Landsat 8 and Sentinel-2 surface reflectance and environmental indices (in addition to LST and topographic variables derived from a single source). The R^2^ values for all models were relatively low (<0.20), indicating that environmental variables alone were not able to describe more than 15–20% of the variability in *S*. *haematobium* prevalence, regardless of RS data source or extraction mask. The predicted prevalence at the pixel level ranged from approximately 5% to 28% (Fig 4). Aggregated predicted community-level prevalence ranged between 7% and 22%, as compared to the observed prevalence range of 0–40%.

**Table 4:** Results of environmental models for various extraction masks showing the R^2^ value and Spearman’s rank correlation value (r) between model predicted and observed prevalence values.

Mask*	R^2^ (RMSE)	r (p-value)	R^2^ (RMSE)	r (p-value)
Landsat 8 data			Sentinel-2 data	
None {1}	0.14 (9.36)	0.49 (< 0.01)	0.14 (9.65)	0.33 (< 0.01)
Unpopulated {2}	0.17 (9.07)	0.46 (< 0.01)	0.14 (9.80)	0.28 (< 0.01)
All WBs {4}	0.15 (9.62)	0.48 (< 0.01)	0.14 (9.70)	0.40 (< 0.01)
Unmined WBs {5}	0.12 (9.68)	0.51 (< 0.01)	0.12 (9.71)	0.34 (< 0.01)
SWAPs {6}	0.15 (9.47)	0.76 (< 0.01)	0.13 (9.43)	0.67 (< 0.01)

* The number in brackets refers to the mask in Fig 3

**Fig 5:**
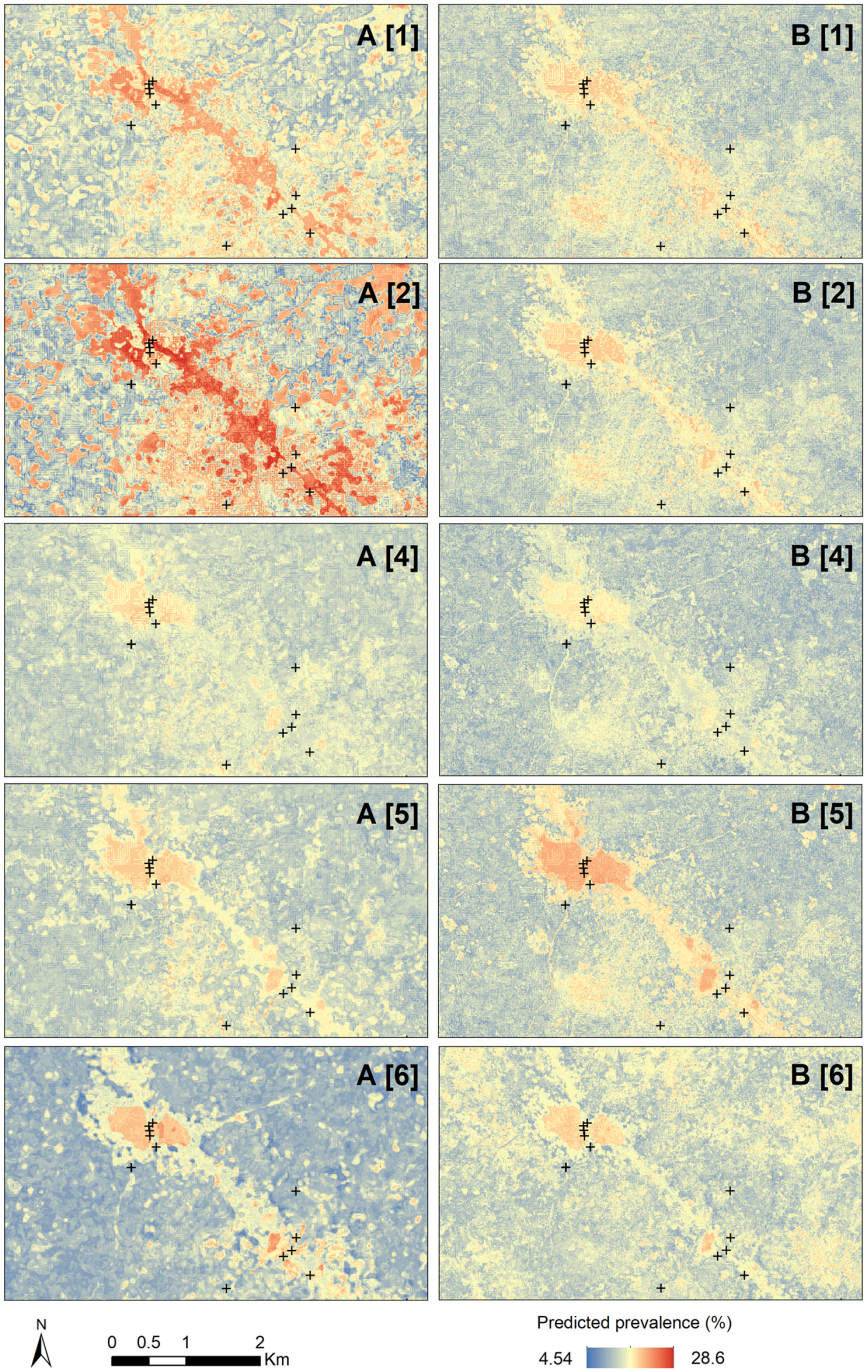
Predicted prevalence from Landsat 8 data (A) and Sentinel-2 data (B) for five extraction masks {1, 2, 4, 5, and 6}. Surface water access points are shown as + symbols. The scale and extent of the image match Fig 4(B).

Correlations between observed and predicted prevalence values were higher on average for models produced using Landsat 8 environmental data as compared to Sentinel-2 data (both in combination with LST and topographic variables). Models derived using the SWAP mask produced the highest correlation values using both Landsat 8 (r = 0.76, p < 0.01) and Sentinel-2 data (r = 0.67, p < 0.01) (Table 4). However, scatter plots of observed versus predicted values still deviated substantially from the line of equality (S22 and S23 Figs, Supporting Information) due to the overall low R^2^ values. From a visual assessment of the predicted prevalence surfaces produced using environmental variables (Fig 5; S24–S33 Figs, Supporting Information), it appears that the SWAP mask resulted in more precise prediction, including correct delineation of water bodies as high-risk locations (Fig 5, panel A6).

Variable importance was also explored using the IncNodePurity measure from random forest models (S22 and S23 Figs, Supporting Information). MNDWI was an important water index, particularly when environmental data were extracted without knowledge of water contact sites (masks 1, 2, 4, and 5). Vegetation indices were not commonly observed among the top five important variables in the Landsat 8 models; EVI and NDVI were the most important vegetation indices in the Sentinel-2 models. Slope and elevation were important in many models, whereas stream order was always the least important variable.

### Contribution of WASH variables

**Fig 6:**
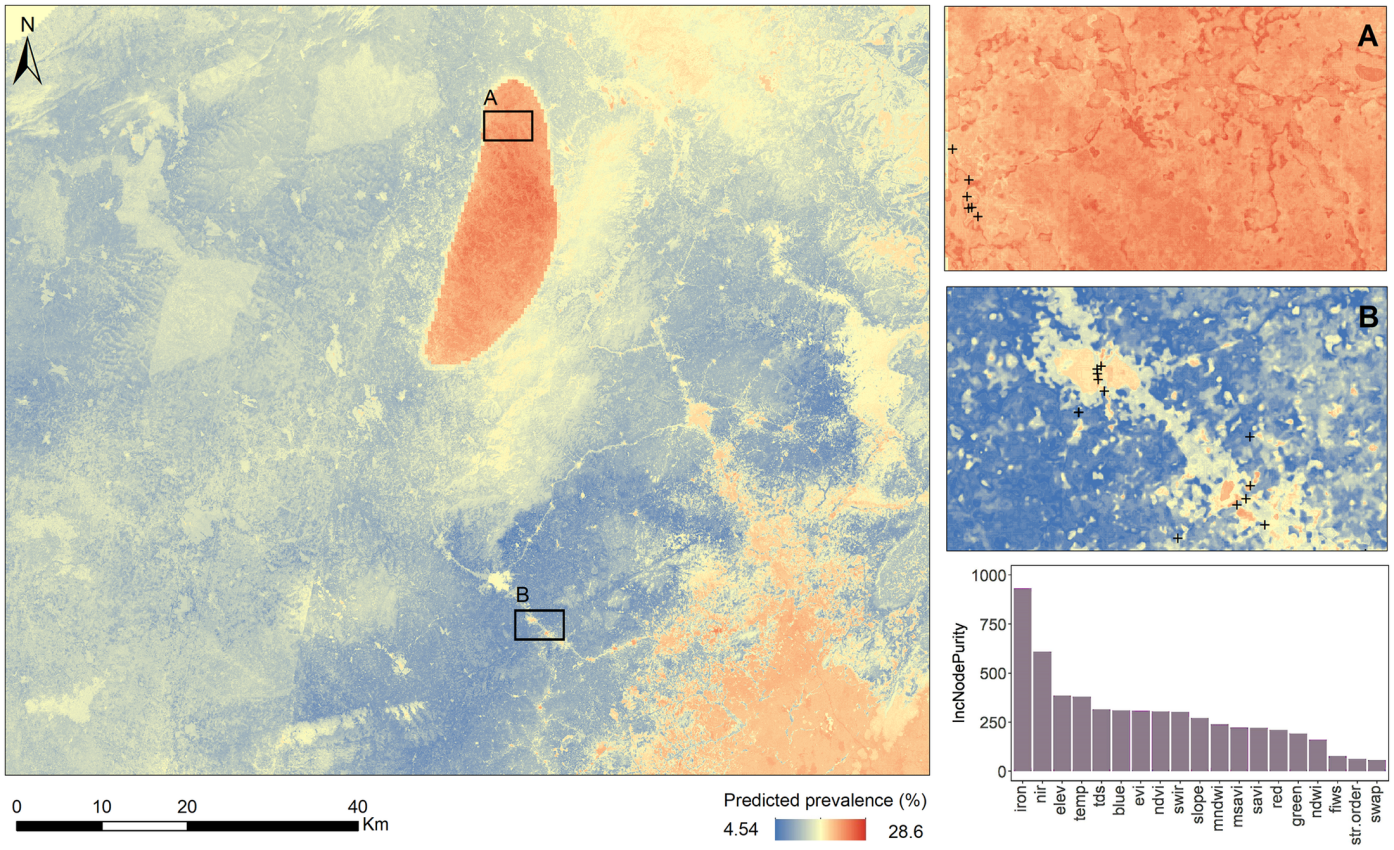
Predicted prevalence for the entire study area; for two smaller zoom windows (A and B); and variable importance values for the final model conducted with Landsat 8 environmental, topographic, and WASH variables. The scales and extents of A and B match Figs 4 and 5. Surface water access points are shown as + symbols.

The final model consisted of environmental variables derived from Landsat 8 data using the SWAP mask in combination with WASH variables. The addition of WASH variables only slightly increased the R^2^ value from 0.15 to 0.17 and decreased the RMSE from 9.47 to 9.03. However, iron concentration became by far the most important variable. The importance of iron concentration was also evident in the predicted prevalence surfaces, with high values on the western side of the Atiwa Mountain Range (Fig 6) coinciding with high groundwater iron content (S21 Fig, Supporting Information). FIWS and SWAP access indicators were not important in the final model. Of the environmental variables, elevation remained important and stream order remained unimportant (Fig 6). The correlations between predicted and observed values were not extracted for the final model because multiple masks were used in the model.

## Discussion

In this study, we utilized publicly available environmental data from two multispectral optical sensors in combination with topographic variables and field-collected WASH variables to assess their performance in predicting *S*. *haematobium* prevalence at a sub-national spatial extent. Furthermore, we tested five methods of environmental data extraction with varying degrees of ecologic relevance. In epidemiologic literature, schistosomiasis is known as a focal disease, meaning that neighboring villages with seemingly similar conditions can have drastically different transmission profiles and disease prevalence levels [10,18,19]. This study attempted to characterize some of the sources of spatial heterogeneity at small spatial extents using fine resolution RS data and WASH-related risk factors.

We found that knowledge of water contact sites shows promise in schistosomiasis risk prediction at small spatial extents. According to a visual assessment, environmental data extracted using the SWAP mask more precisely delineated water bodies as high-risk locations within communities (Fig 5). This mask also produced the highest correlation between model predicted and observed prevalence values, depicting heterogeneity in transmission risk among communities (Table 4).

Of the two water indices we explored, MNDWI was the preferred index due to more accurate detection of water bodies. NDWI values were equally high for water and developed pixels (roads and settled areas), indicating false detection of water bodies. Generally, higher values of MNDWI correlated with higher schistosomiasis risk. However, even MNDWI could not detect small streams that sustained most of surface water use (i.e., SWAPs). Further investigation of these two indices and their utility in water-related disease modeling is recommended. Vegetation indices did not play a major role in prediction. This is not surprising, especially in the SWAP mask models, as these indices are likely characterizing land vegetation cover, rather than aquatic vegetation that affects intermediate host snail abundance [11].

LST did not exhibit a strong influence on schistosomiasis risk, most probably due to the lack of variability in LST values (25–32 °C), all of which were well within the favorable temperature range for snail and cercariae survival [54,55]. Furthermore, because the water bodies in the study area are very small, the spatial resolution of the temperature data (100 m) was likely too coarse to detect water temperature.

Slope and elevation were important in prediction. Higher elevation correlated with higher schistosomiasis risk, counter to the literature, likely because the Atiwa Mountain Range is still quite low in elevation, far below the 2,000-m above sea level threshold for *S*. *haematobium* transmission [18]. Higher slope correlated with lower schistosomiasis risk, potentially due to faster stream flows. At water velocities > 0.3 m/s, snails can become dislodged and swept away [55]. Surprisingly, stream order was consistently the least important variable in all models, while it demonstrated a significant positive association with schistosomiasis risk in other studies [17,22]. A potential explanation for this is the abundance of small streams throughout the study communities, widespread preference of people for surface water over groundwater, and hence their uniform extensive use.

In our study, variables of improved and unimproved water access were not predictive of schistosomiasis risk, consistently with the findings of Lai et al. [4]. However, high iron concentration in groundwater was associated with increased schistosomiasis risk. Our prior studies have provided qualitative support for the hypothesis that unfavorable groundwater quality in improved water sources (i.e., boreholes and piped water systems) for drinking and laundry is a significant driver of increased surface water use, serving as an indirect risk factor for schistosomiasis transmission. The final model results confirmed this hypothesis, with groundwater iron content being the predominant schistosomiasis risk factor with a much higher IncNodePurity value as compared to any of the environmental variables (Fig 6). Indeed, in Fig 6, the area with high predicted schistosomiasis prevalence in the center of the image corresponds to the high iron concentration cluster (S21 Fig, Supporting Information).

Overall, the models had relatively low predictive power and predicted prevalence values deviated substantially from the observed values, indicating overprediction in the low-prevalence range and underprediction in the high-prevalence range. This is most likely due to the effects of preventive chemotherapy on prevalence measures. With increased treatment frequency, it becomes difficult to detect the effects of environmental conditions on transmission risk [4,22]. It would be valuable to apply these approaches in similar geographic extents with a wider prevalence range. Exploring different methods of defining “communities” over which risk factor variables are aggregated (e.g., varying the buffer radius within which transmission occurs) in other geographic, demographic, and cultural contexts is also recommended.

We also found that Landsat 8 and Sentinel-2 sensors with similar radiometric resolutions (12-bit) and acquisition dates (all images were acquired within one week), on average, had similar predictive capacities. Cloud cover presented a substantial challenge in RS data acquisition from both data sources, with few cloud-free images available only in the dry season (December and January). Additionally, Landsat 8 data were more affected by haze and ocean spray, as compared to Sentinel-2 data. As RS data algorithms improve, future studies should consider repeating the same environmental models using RS data representative of both dry and rainy seasons to analyze the impact of water stability and dynamics. Synthetic Aperture Radar (SAR) data (e.g., from Sentinel-1A) could provide additional information in this and similar cloud-affected regions.

Apart from technical challenges associated with using RS data, several logistic challenges may have affected the quality of this study. First, low attendance in some of the study schools (range 46–95%) associated with sporting events and market days may have affected the prevalence measures. For example, children from agrarian families who were absent on market days are likely different in terms of socioeconomic status and schistosomiasis exposure profile from those who were present and participated in the study. In a smaller study, it would have been possible to go back and screen absentees; in the present study, this was not possible due to time and scheduling limitations and absence of identifying information about participants. Additional challenges arose from working across 10 administrative districts, especially with securing local GHS personnel to administer praziquantel. Scheduling and coordination efforts were further complicated by the community health workers being on strike in some of the districts during the study.

Despite the challenges and limitations, our study makes important contributions to the modeling approaches of schistosomiasis transmission at small spatial extents. First, knowledge of human water contact sites bridges the gap between where prevalence is measured and where transmission may have occurred. This is a critical gap in models that utilize environmental data as predictors of human infection. Second, the impact of groundwater iron concentration on schistosomiasis risk. With prevalence rates up to 40% only six months after preventive chemotherapy and very high rates of fetching surface water (up to 100%) and swimming (up to 90%) [49], reinfection is a major concern in the study area. Groundwater quality in improved water sources, more so than improved water access in general, plays a major role in reinfection patterns and can impede schistosomiasis control. While it is well-established that preventive chemotherapy reduces prevalence and worm burden in the short term, with rapid reinfection, it cannot have more than a temporary effect on transmission without complementary improvements in WASH [23,24,56]. Our extensive experience in the Eastern region of Ghana suggests that it is not only increasing access to WASH resources that matters, but rather increasing utilization of these resources in accordance with local perceptions and preferences. Considering WASH-related risk factors in schistosomiasis prediction can help shift the focus of control strategies from treating symptoms to reducing exposure [56].

## Supporting information

S1 Table Microhematuria prevalence survey results. (XLSX)

S2 Table Spearman’s rank correlations among six environmental indices (all are statistically significant; p < 0.05); Sentinel-2 values are shown in top and Landsat 8 in bottom of the matrix. (XLSX)

S1 Fig Data processing steps. (TIF)

S2 Fig Data analysis steps. (TIF)

S3 Fig Sentinel-2 blue band reflectance values. (TIF)

S4 Fig Sentinel-2 green band reflectance values. (TIF)

S5 Fig Sentinel-2 red band reflectance values. (TIF)

S6 Fig Sentinel-2 near infrared band reflectance values. (TIF)

S7 Fig Sentinel-2 short wavelength infrared band reflectance values. (TIF)

S8 Fig Landsat 8 land surface temperature values. (TIF)

S9 Fig Sentinel-2 NDVI values. (TIF)

S10 Fig Sentinel-2 EVI values. (TIF)

S11 Fig Sentinel-2 SAVI values. (TIF)

S12 Fig Sentinel-2 MSAVI values. (TIF)

S13 Fig Sentinel-2 NDWI values. (TIF)

S14 Fig Sentinel-2 MNDWI values. (TIF)

S15 Fig Elevation values (in meters) derived from GDEM. (TIF)

S16 Fig Slope values (in degrees) derived from GDEM. (TIF)

S17 Fig Slope values derived from GDEM. (TIF)

S18 Fig Functional improved water source (FIWS) access values derived in ArcGIS from field data. (TIF)

S19 Fig Perennial surface water source (SWAP) access values derived in ArcGIS from field data. (TIF)

S20 Fig Interpolated total dissolved solids (TDS) concentration values (in mg/L) derived in ArcGIS from field data. (TIF)

S21 Fig Interpolated iron concentration values (in mg/l) derived in ArcGIS from field data. (TIF)

S22 Fig Scatter plots of model predicted (x-axis) vs. observed (y-axis) prevalence values as compared to the line of equality [left]; variable importance values [right] for random forest models conducted with environmental variables from Landsat 8 and topographic variables from GDEM. (TIF)

S23 Fig Scatter plots of model predicted (x-axis) vs. observed (y-axis) prevalence values as compared to the line of equality [left]; variable importance values [right] for random forest models conducted with environmental variables from Sentinel-2 and topographic variables from GDEM. (TIF)

S24 Fig Predicted prevalence values using Landsat 8 data (mask 1). (TIF)

S25 Fig Predicted prevalence values using Sentinel-2 data (mask 1). (TIF)

S26 Fig Predicted prevalence values using Landsat 8 data (mask 2). (TIF)

S27 Fig Predicted prevalence values using Sentinel-2 data (mask 2). (TIF)

S28 Fig Predicted prevalence values using Landsat 8 data (mask 4). (TIF)

S29 Fig Predicted prevalence values using Sentinel-2 data (mask 4). (TIF)

S30 Fig Predicted prevalence values using Landsat 8 data (mask 5). (TIF)

S31 Fig Predicted prevalence values using Sentinel-2 data (mask 5). (TIF)

S32 Fig Predicted prevalence values using Landsat 8 data (mask 6). (TIF)

S33 Fig Predicted prevalence values using Sentinel-2 data (mask 6). (TIF)
