# Supplementary figures and images for: Improving spatial prediction of Schistosoma haematobium prevalence in southern Ghana through new remote sensors and local water access profiles
Source: PLoS Negl Trop Dis. 2018 Jun 4;12(6):e0006517. doi: 10.1371/journal.pntd.0006517 (PMC6014678; doi:10.1371/journal.pntd.0006517)

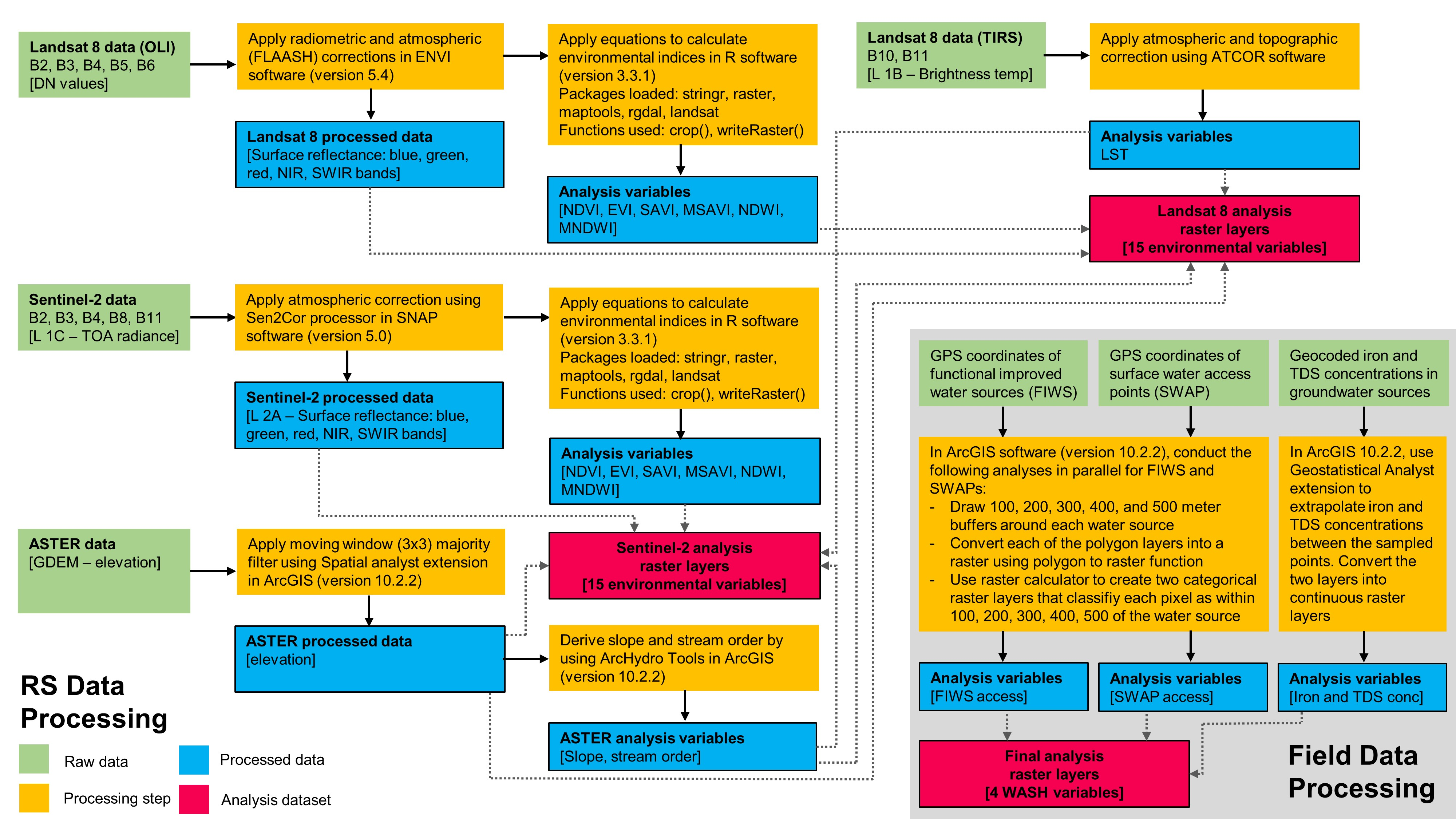

Supplement: S1 Fig — (TIF) [file pntd.0006517.s003.tif]

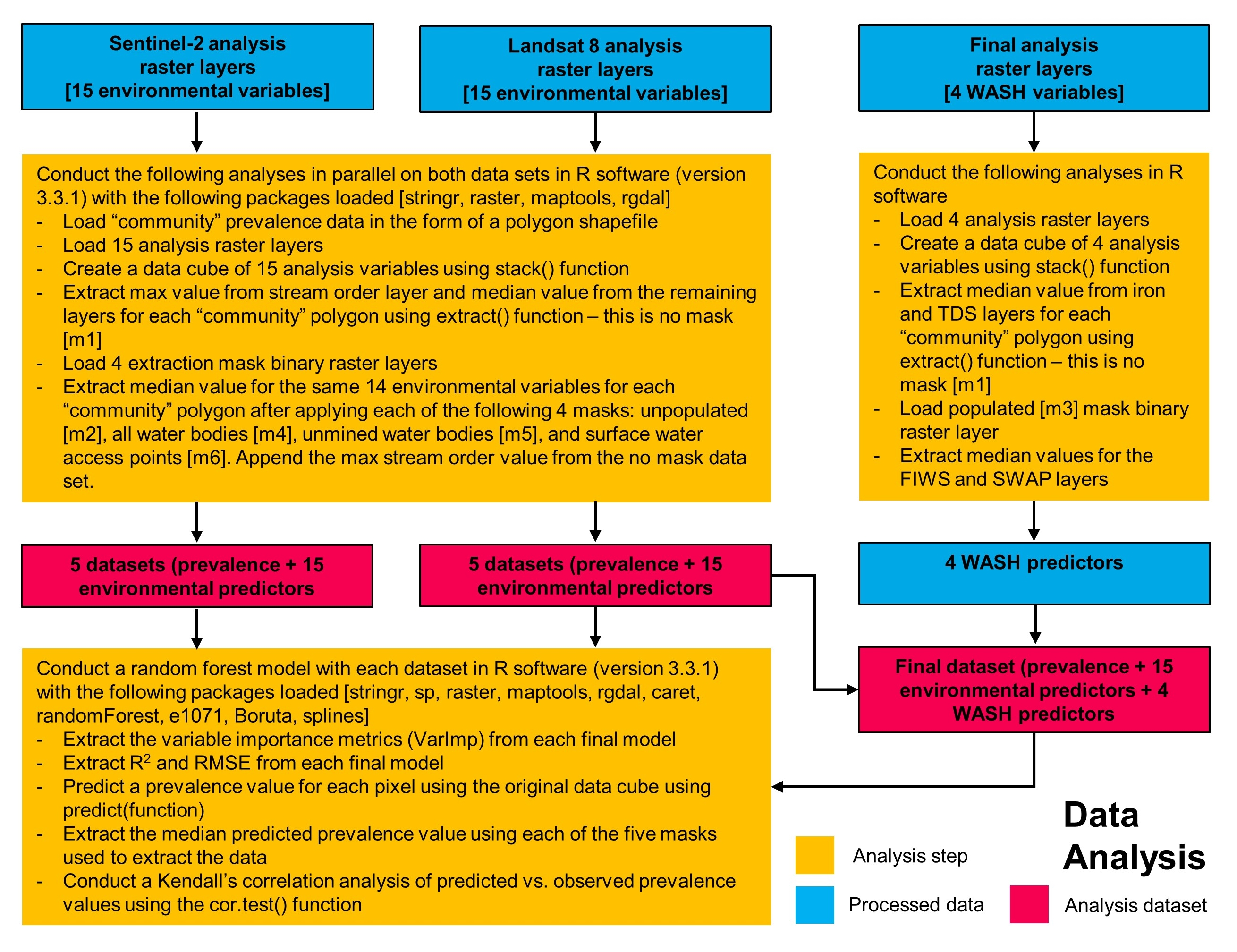

Supplement: S2 Fig — (TIF) [file pntd.0006517.s004.tif]

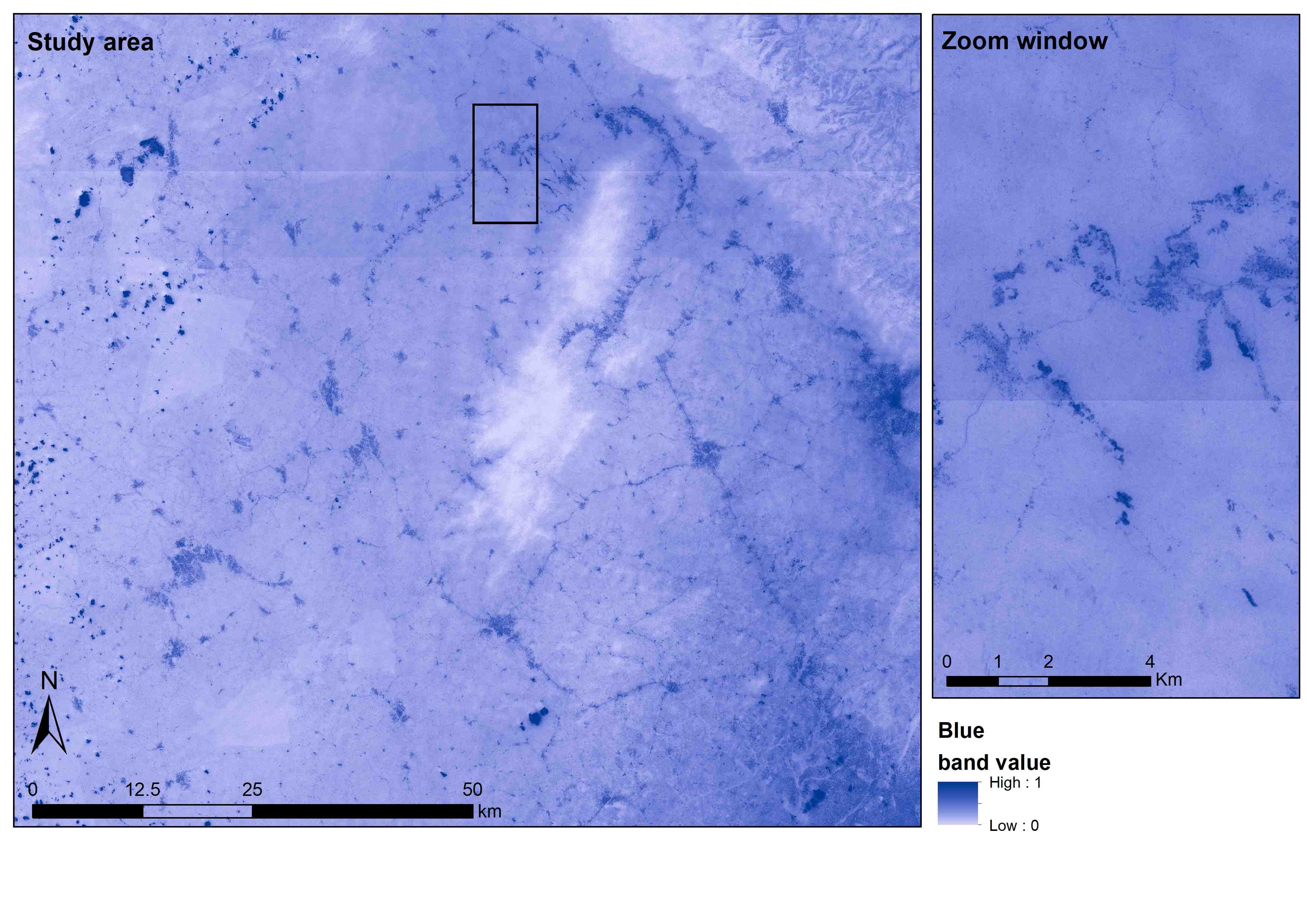

Supplement: S3 Fig — (TIF) [file pntd.0006517.s005.tif]

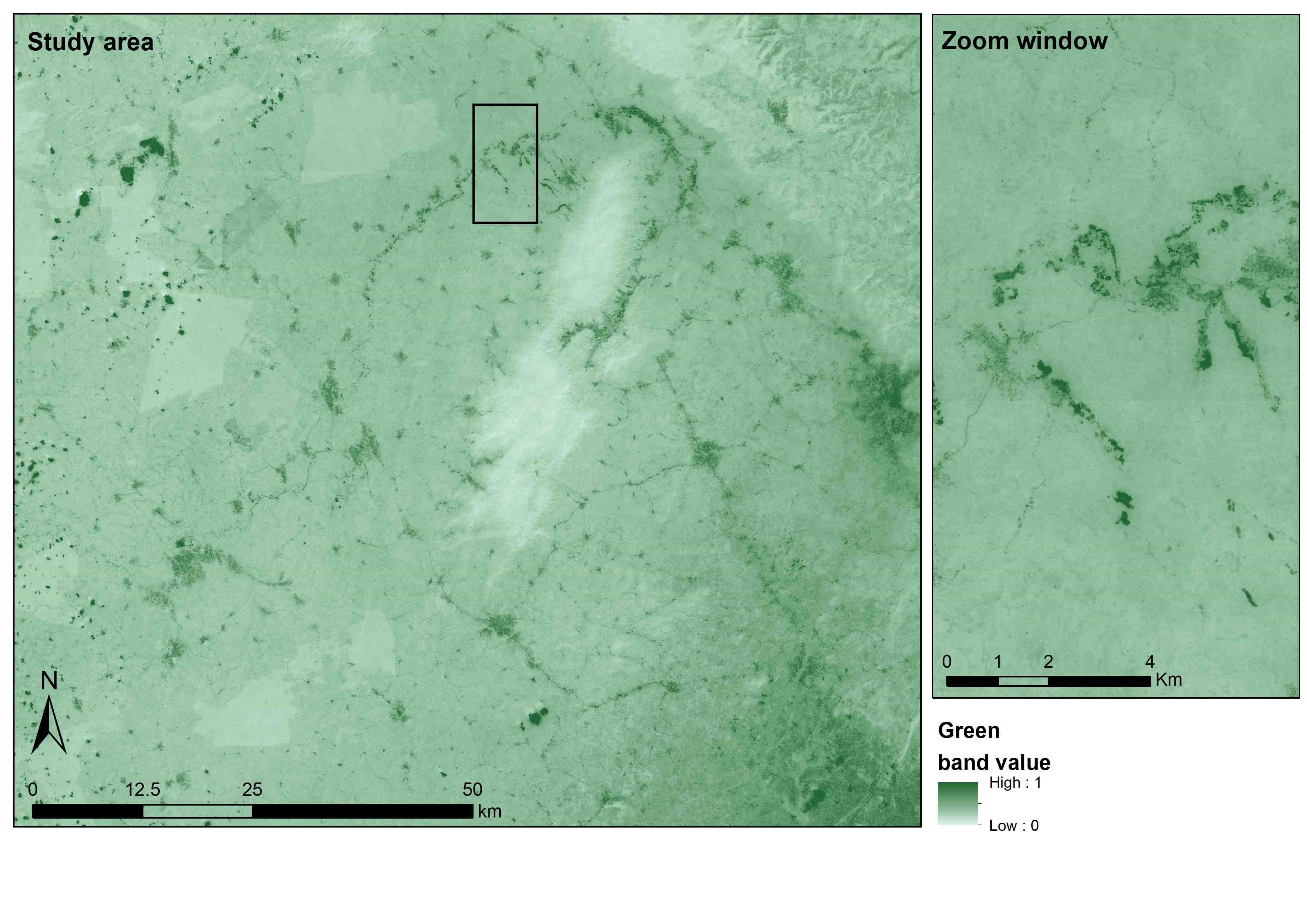

Supplement: S4 Fig — (TIF) [file pntd.0006517.s006.tif]

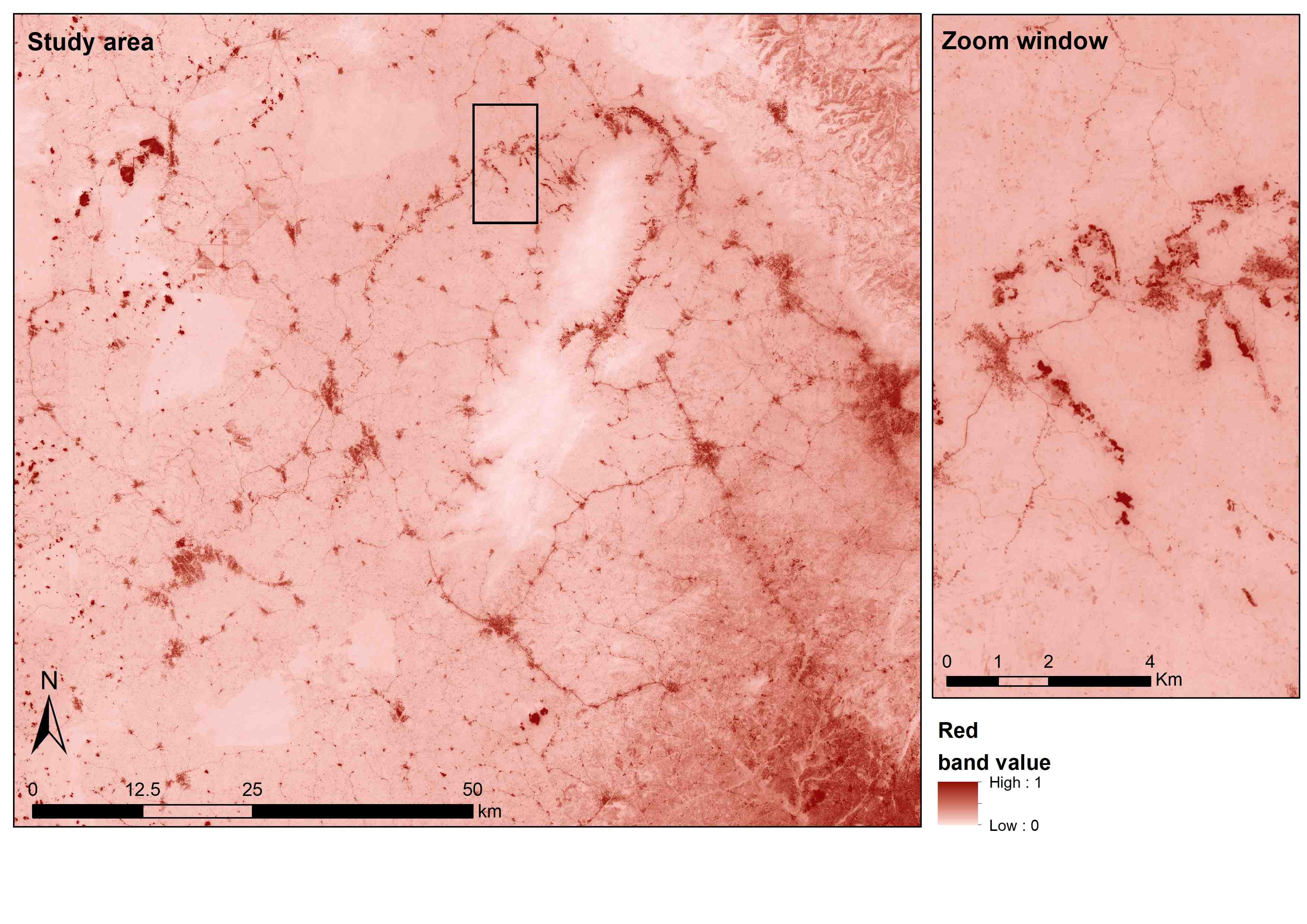

Supplement: S5 Fig — (TIF) [file pntd.0006517.s007.tif]

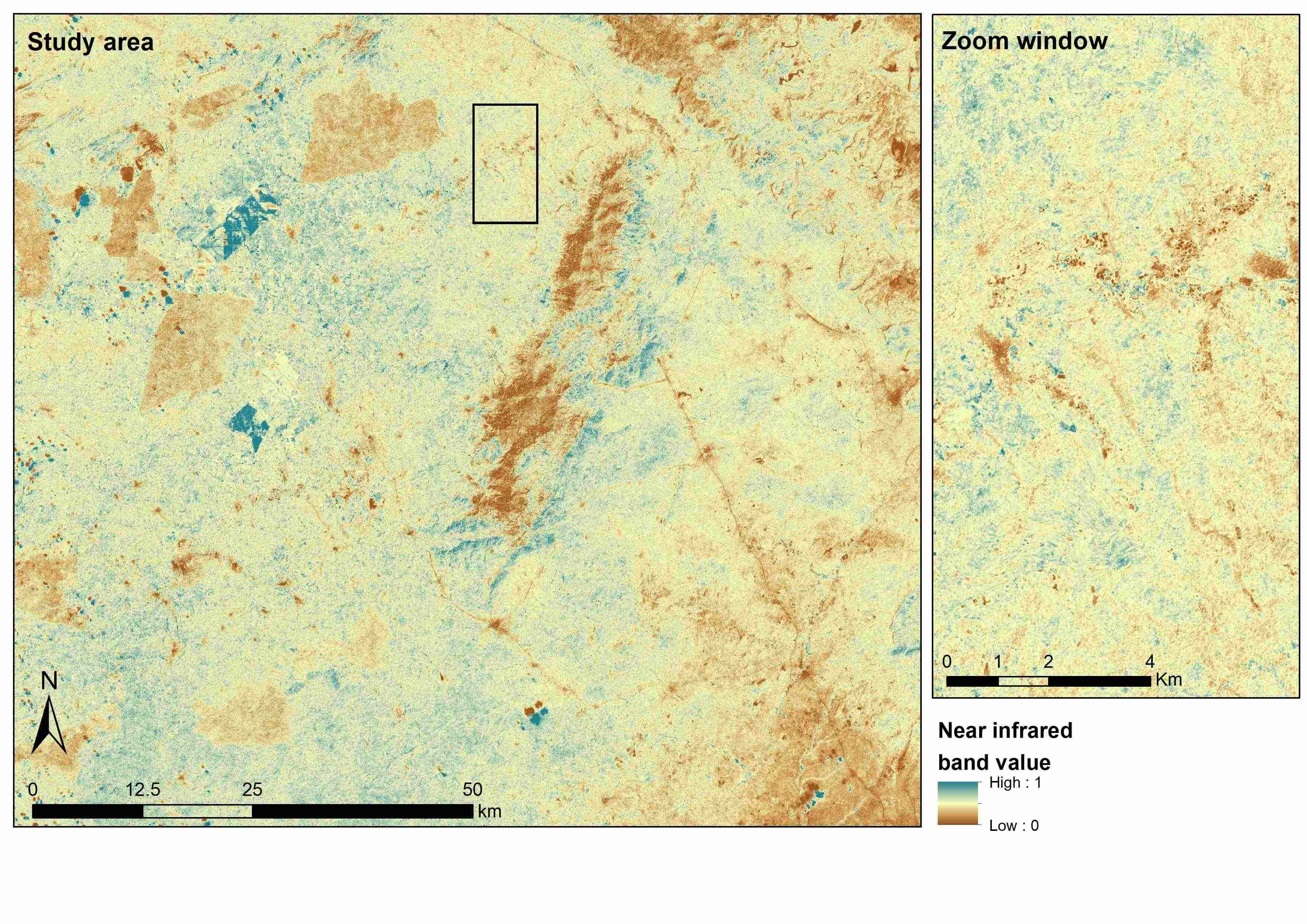

Supplement: S6 Fig — (TIF) [file pntd.0006517.s008.tif]

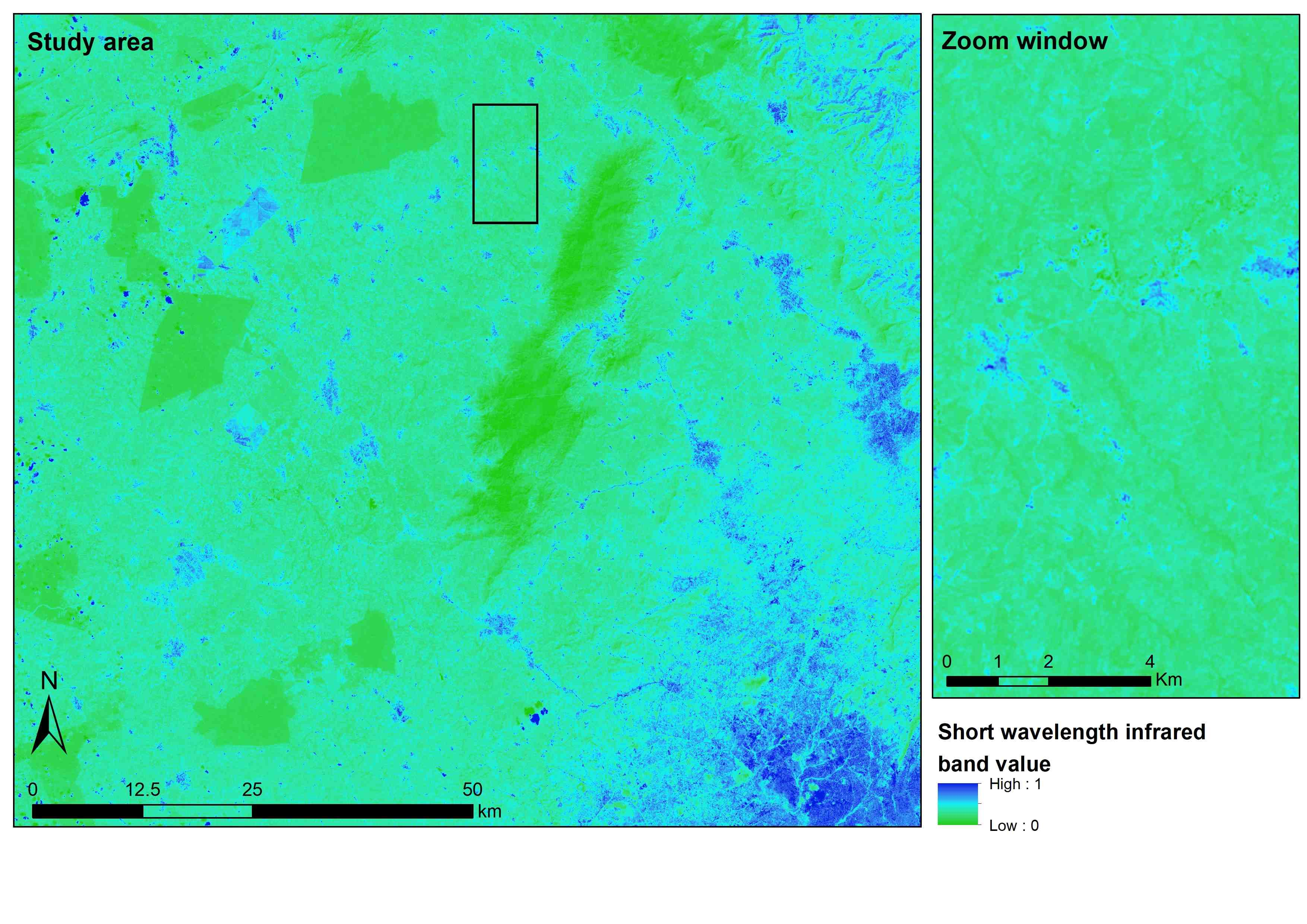

Supplement: S7 Fig — (TIF) [file pntd.0006517.s009.tif]

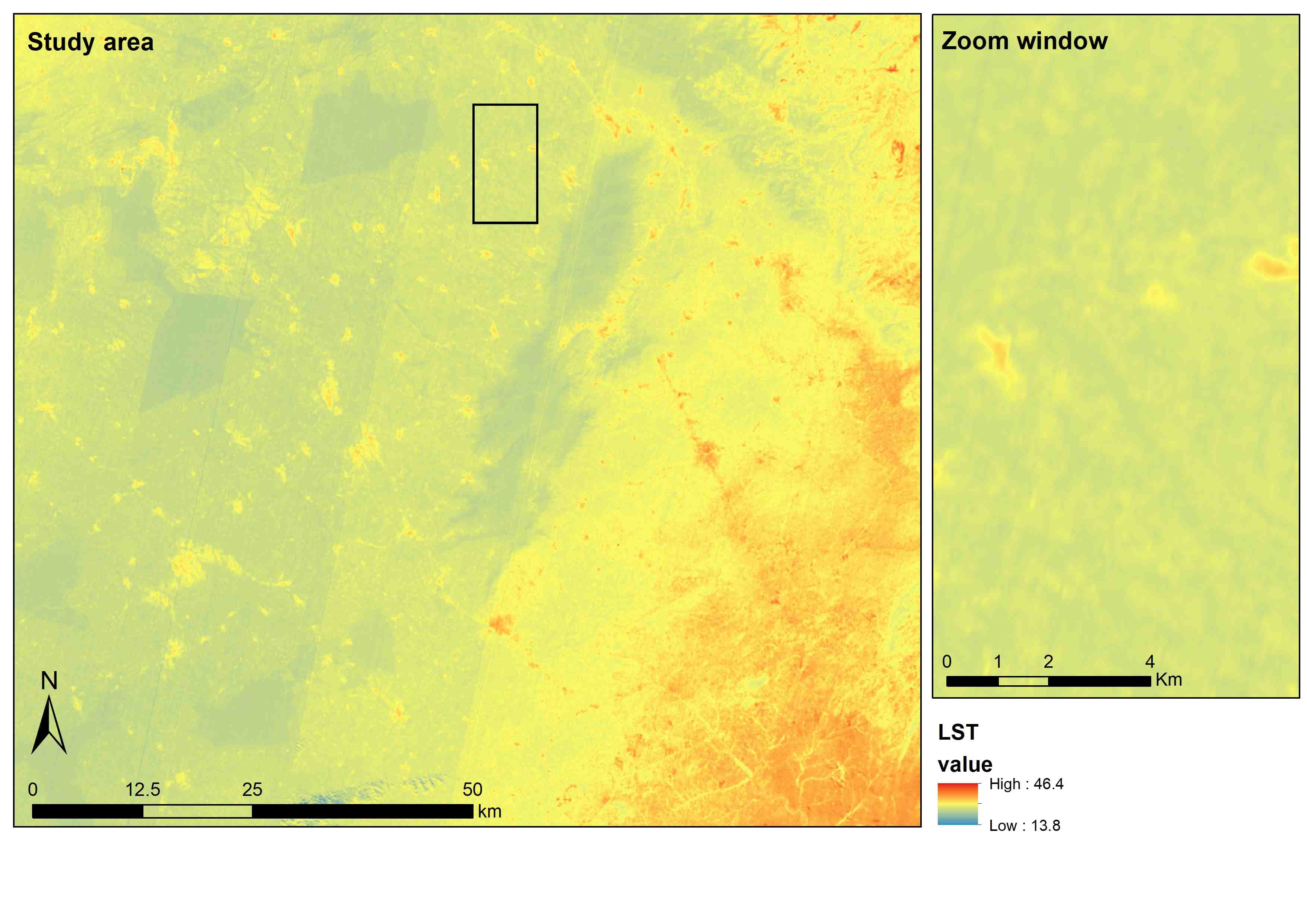

Supplement: S8 Fig — (TIF) [file pntd.0006517.s010.tif]

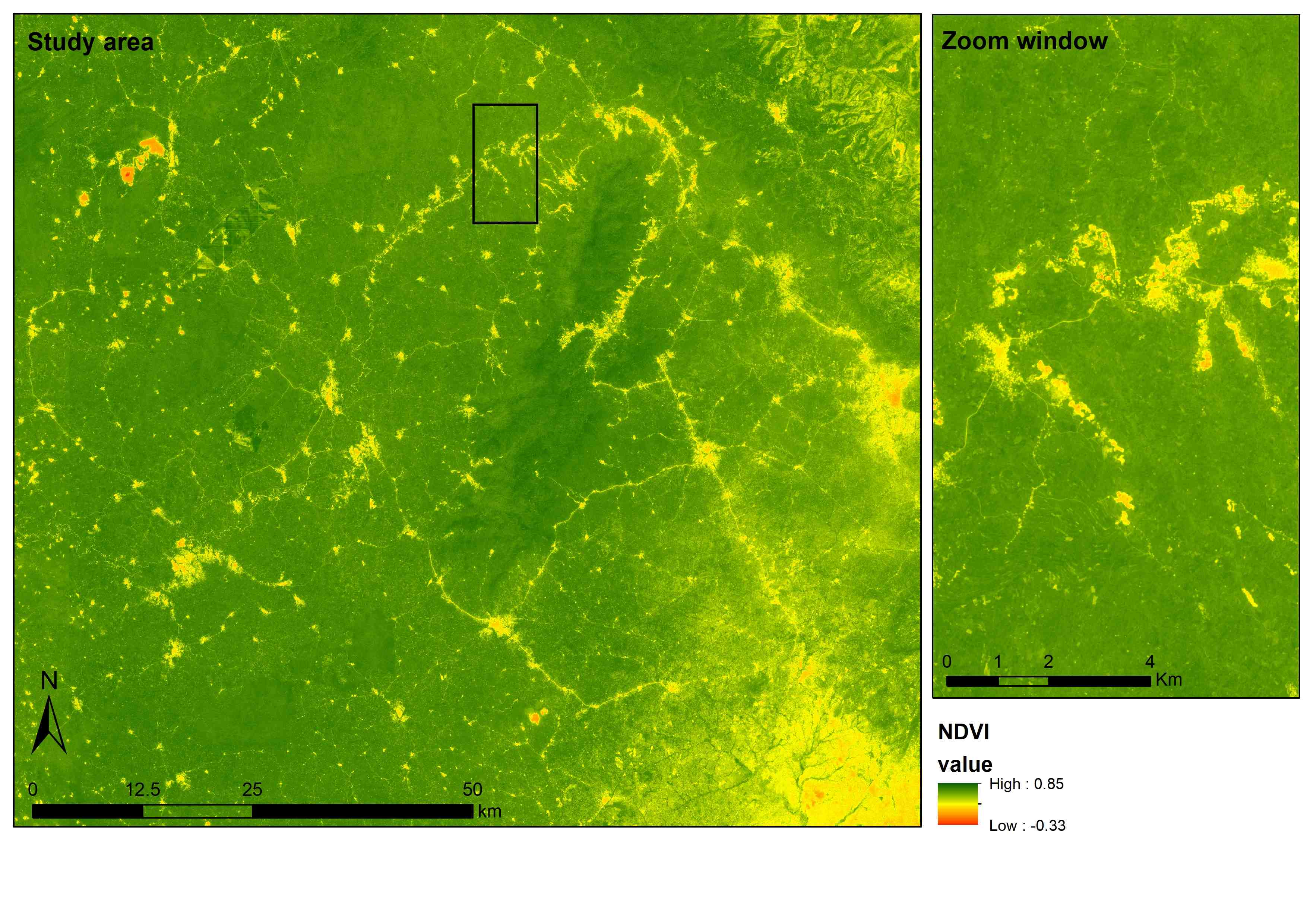

Supplement: S9 Fig — (TIF) [file pntd.0006517.s011.tif]

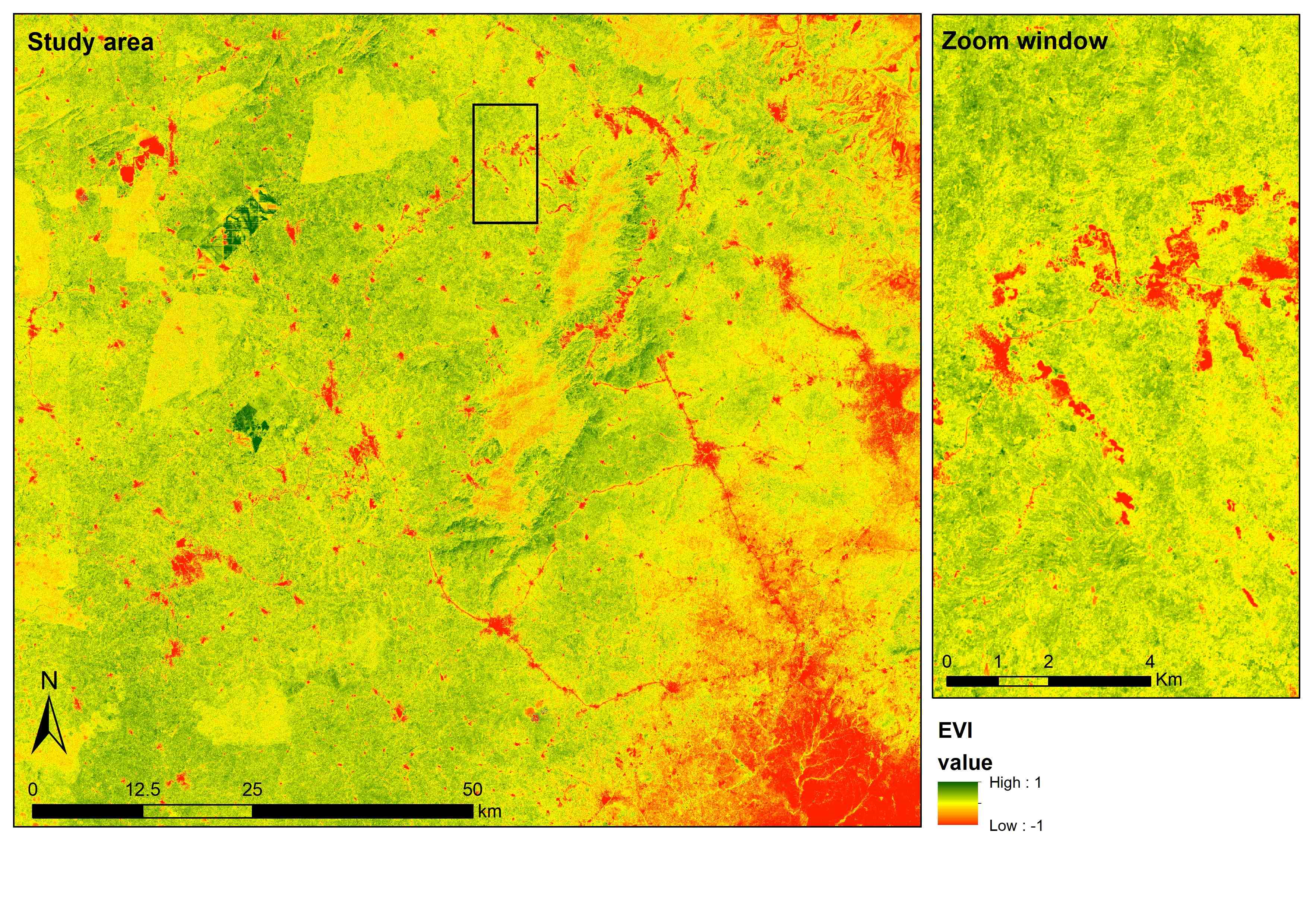

Supplement: S10 Fig — (TIF) [file pntd.0006517.s012.tif]

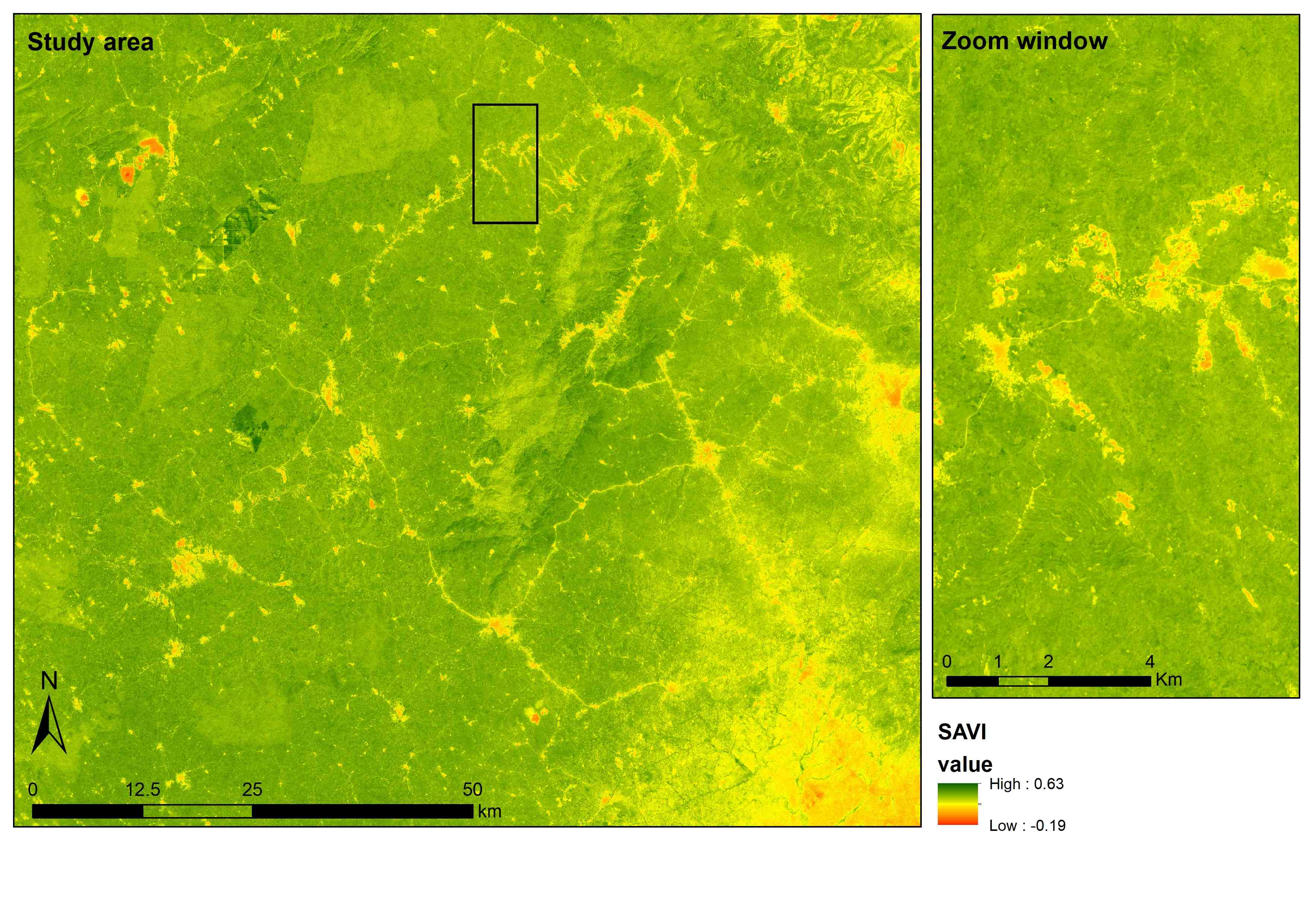

Supplement: S11 Fig — (TIF) [file pntd.0006517.s013.tif]

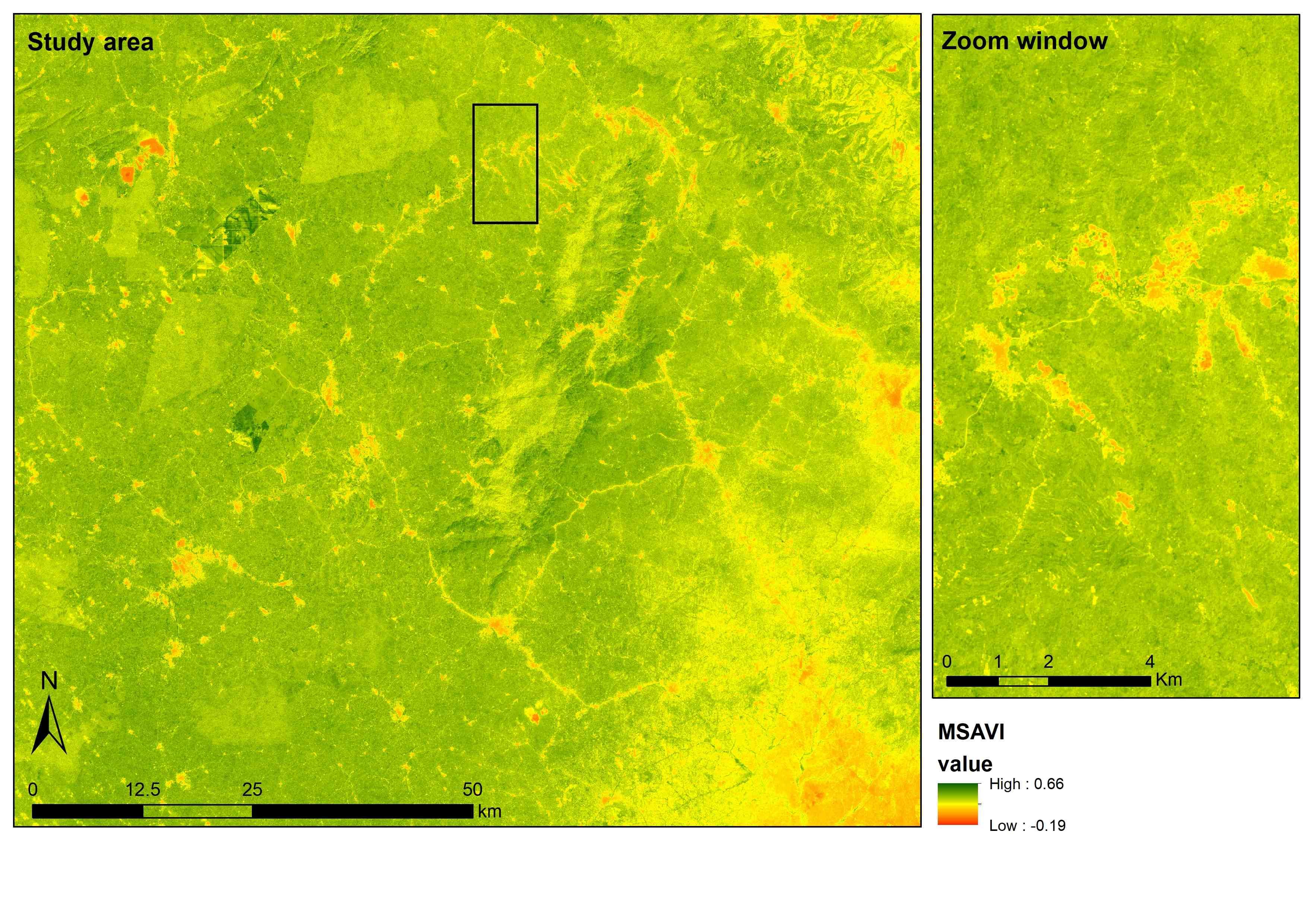

Supplement: S12 Fig — (TIF) [file pntd.0006517.s014.tif]

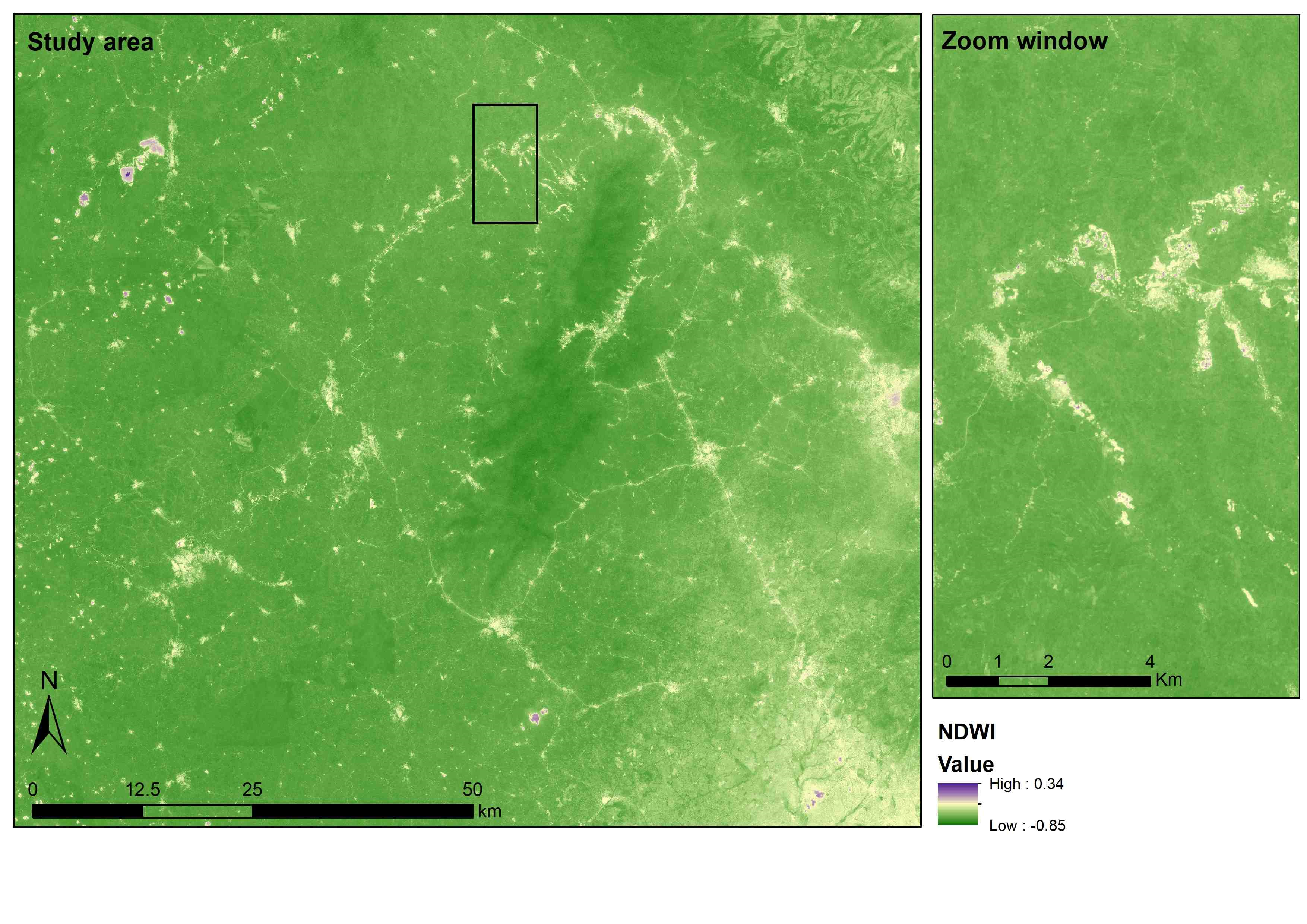

Supplement: S13 Fig — (TIF) [file pntd.0006517.s015.tif]

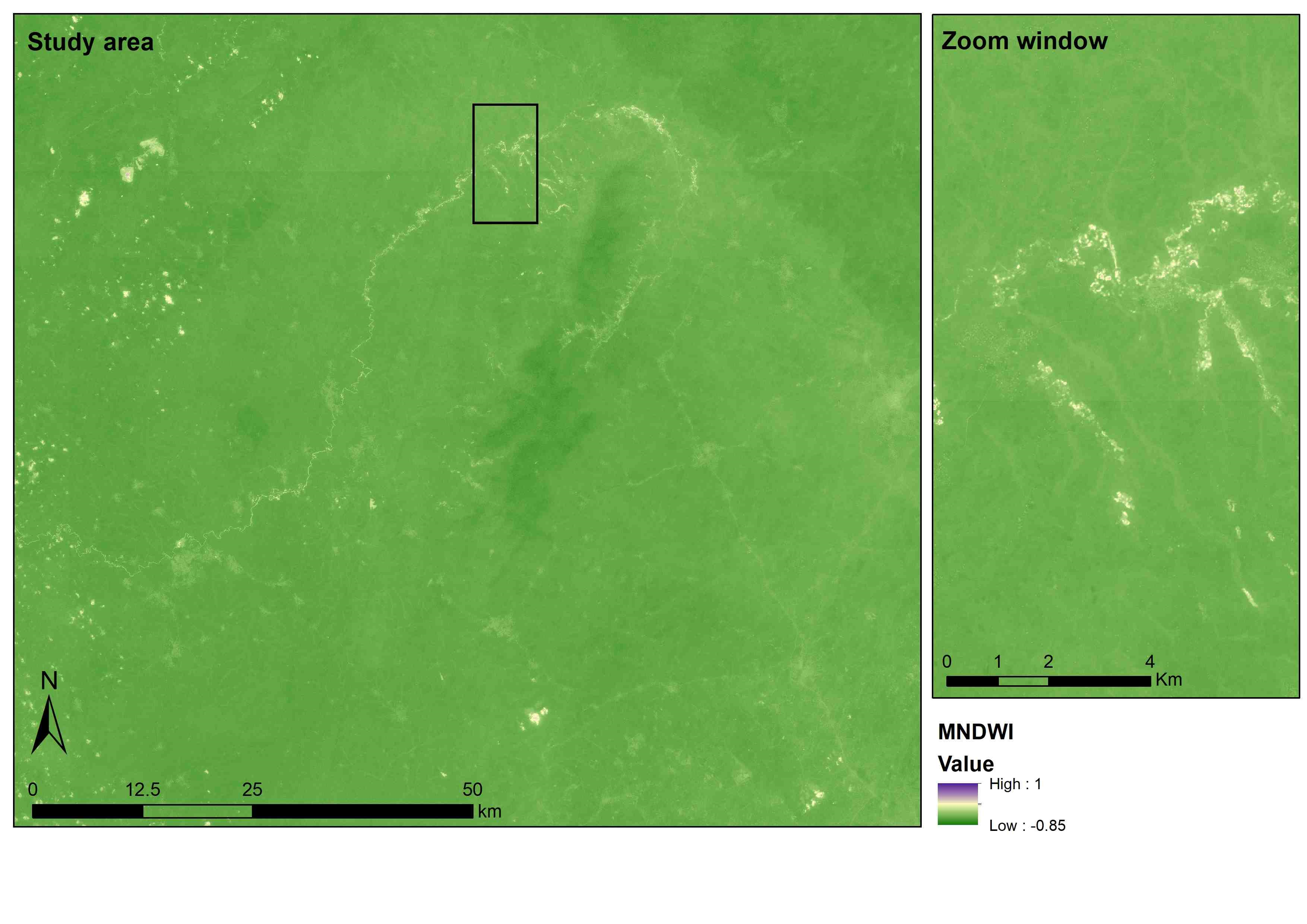

Supplement: S14 Fig — (TIF) [file pntd.0006517.s016.tif]

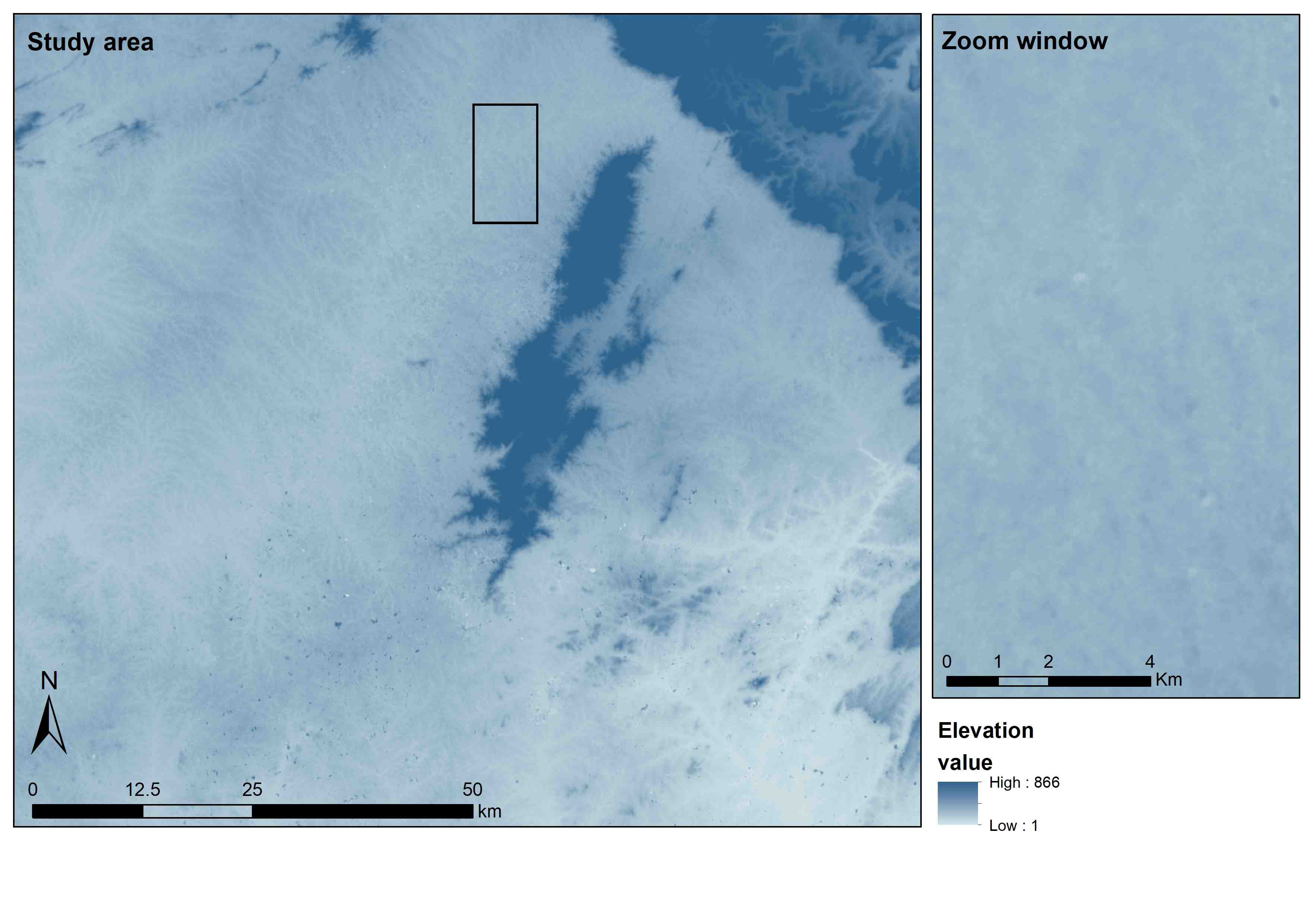

Supplement: S15 Fig — (TIF) [file pntd.0006517.s017.tif]

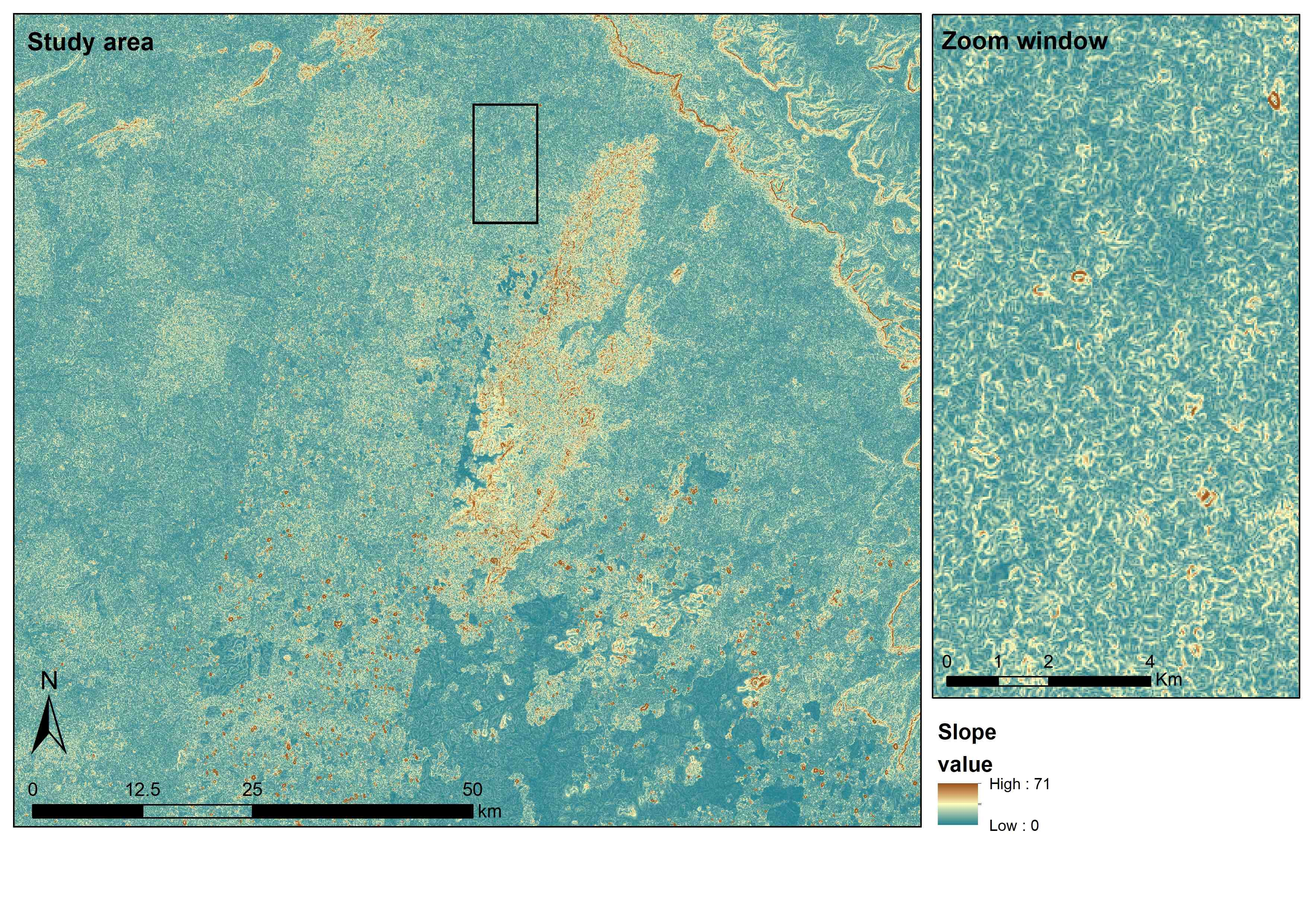

Supplement: S16 Fig — (TIF) [file pntd.0006517.s018.tif]

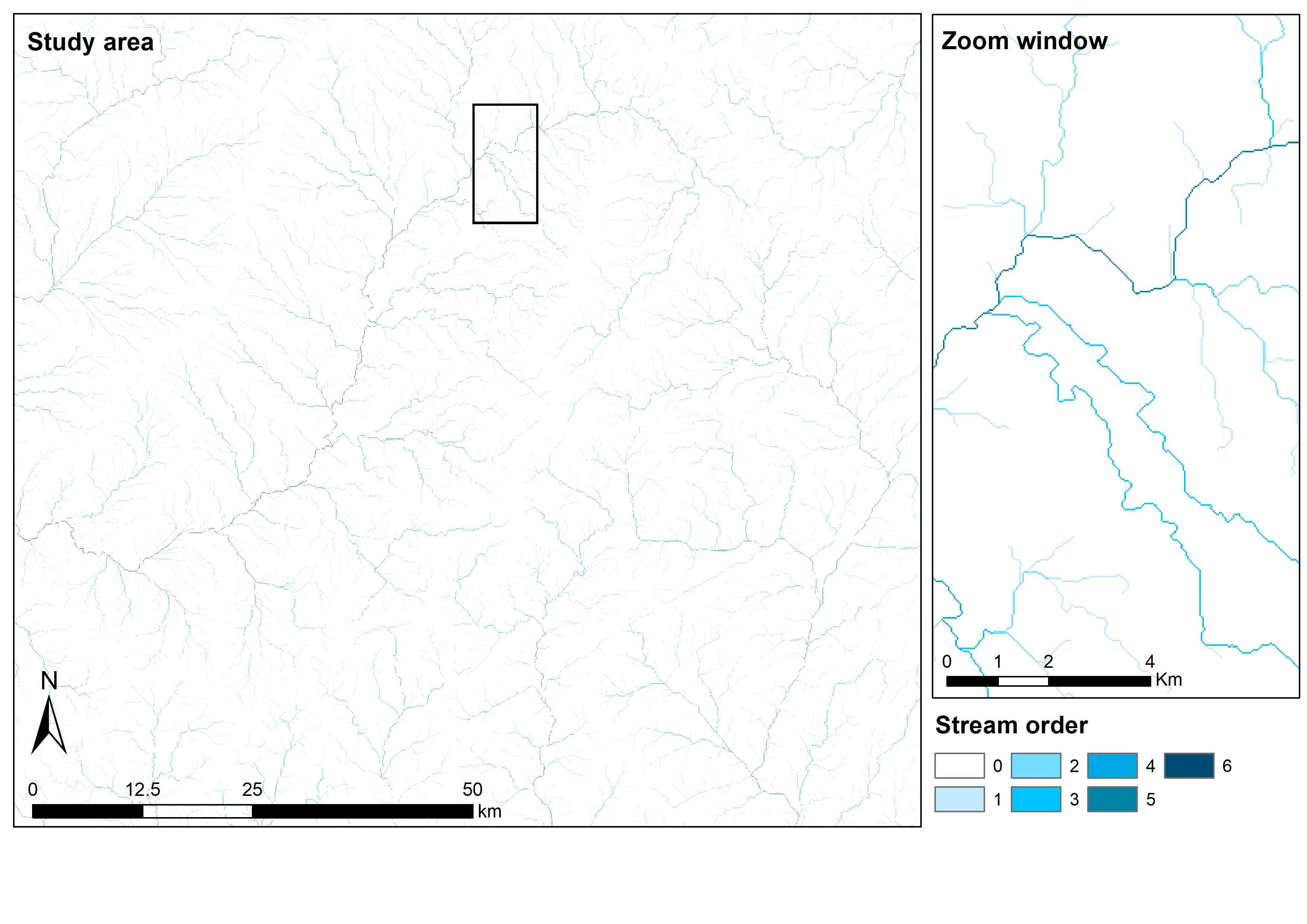

Supplement: S17 Fig — (TIF) [file pntd.0006517.s019.tif]

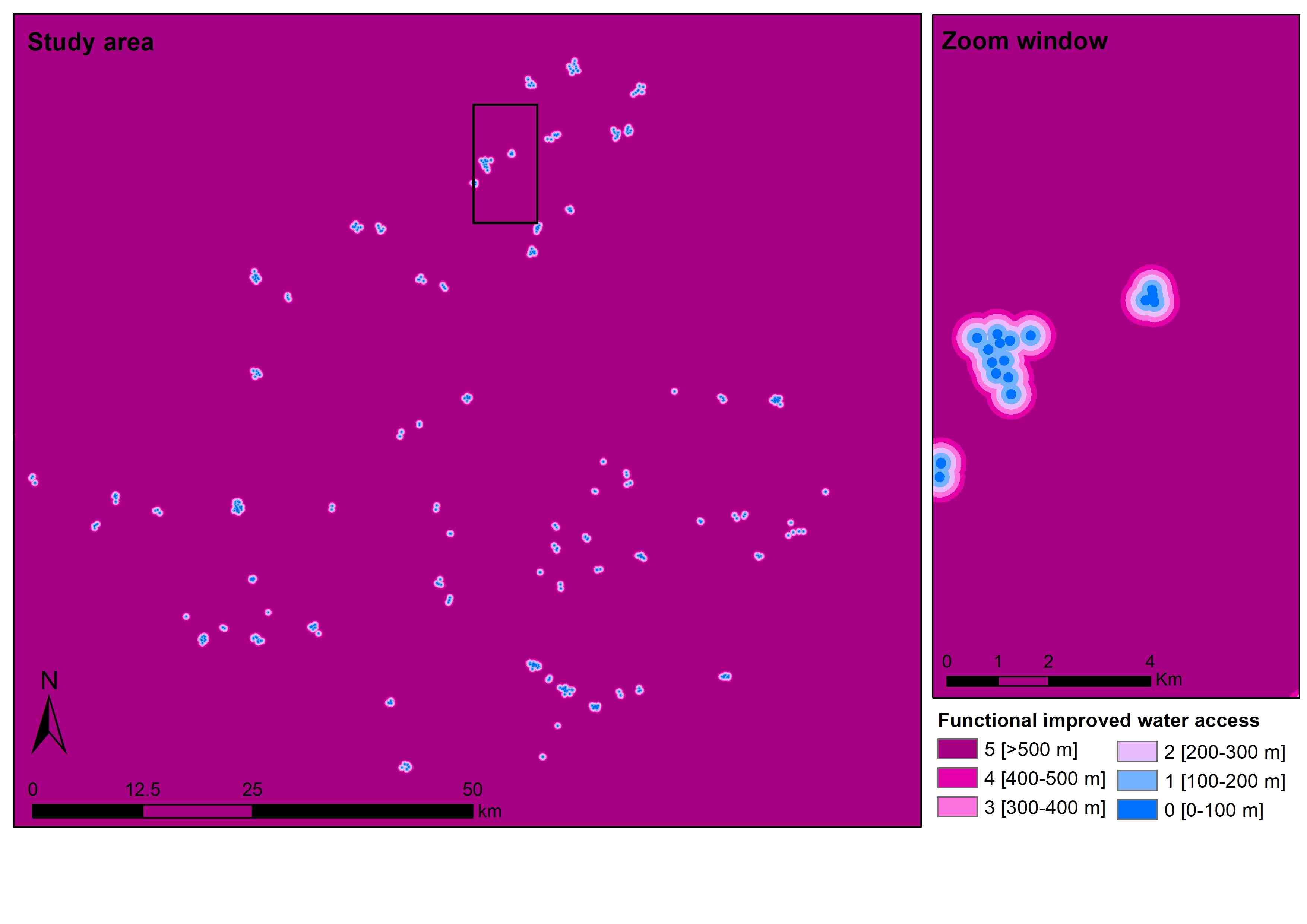

Supplement: S18 Fig — (TIF) [file pntd.0006517.s020.tif]

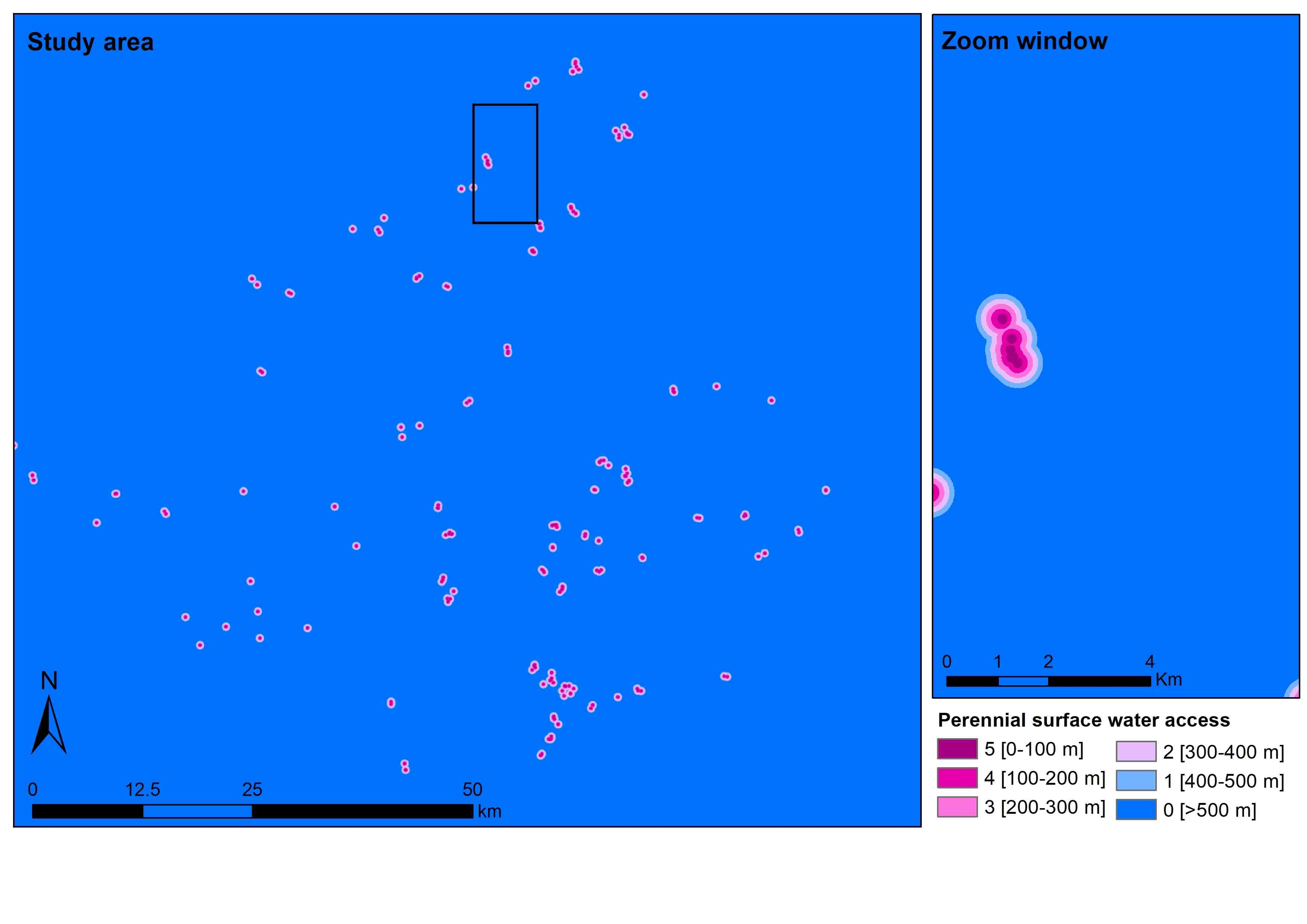

Supplement: S19 Fig — (TIF) [file pntd.0006517.s021.tif]

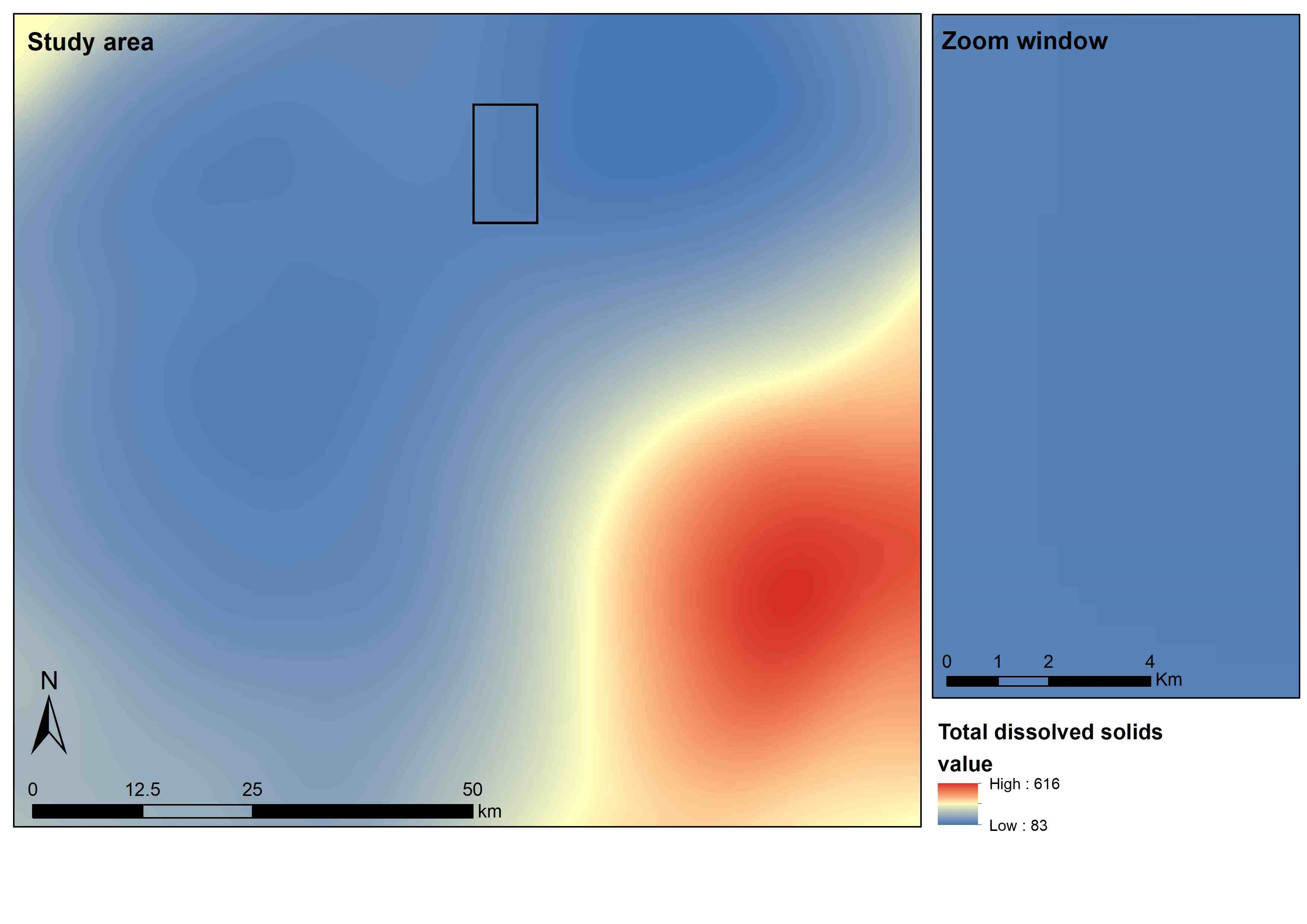

Supplement: S20 Fig — (TIF) [file pntd.0006517.s022.tif]

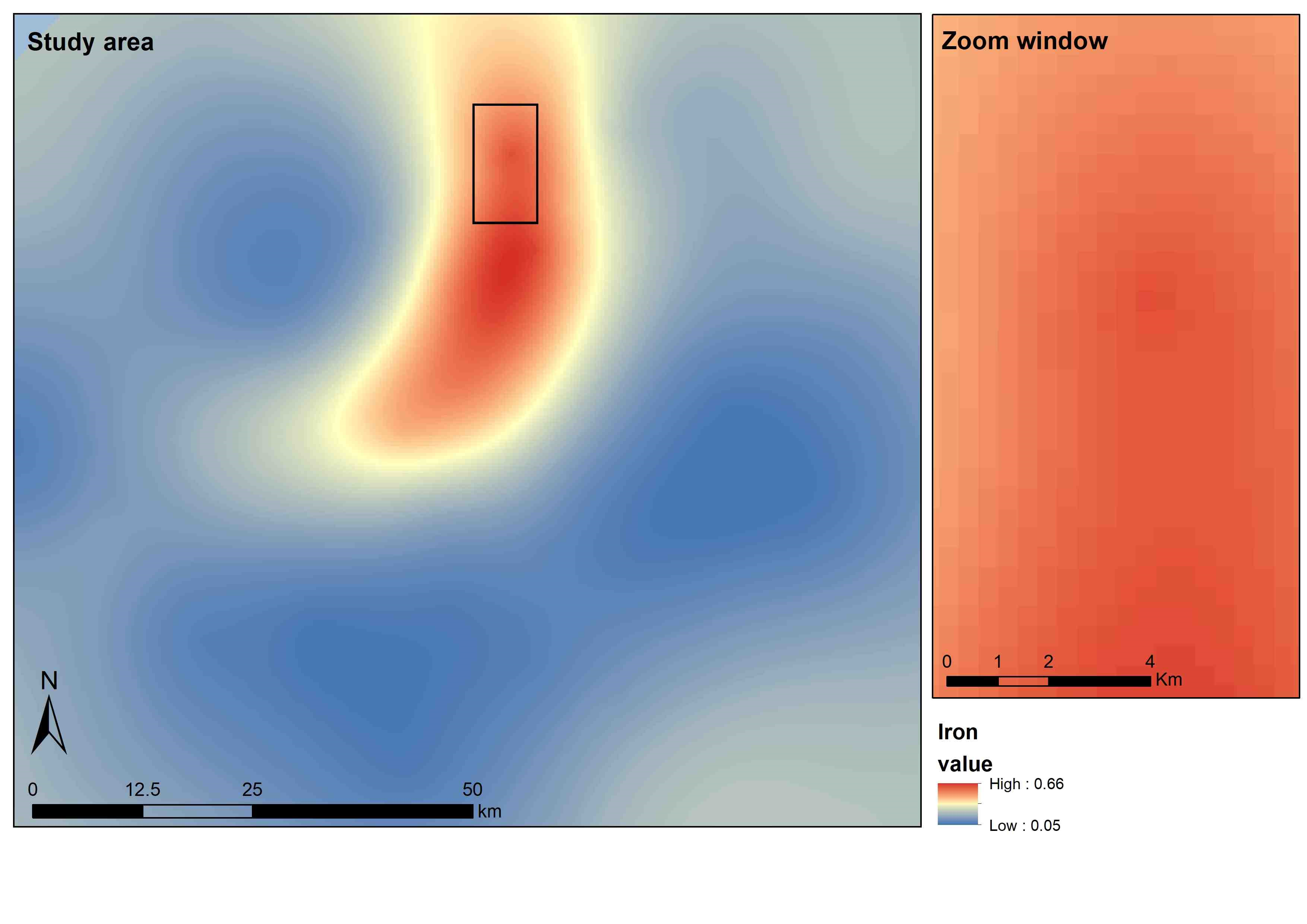

Supplement: S21 Fig — (TIF) [file pntd.0006517.s023.tif]

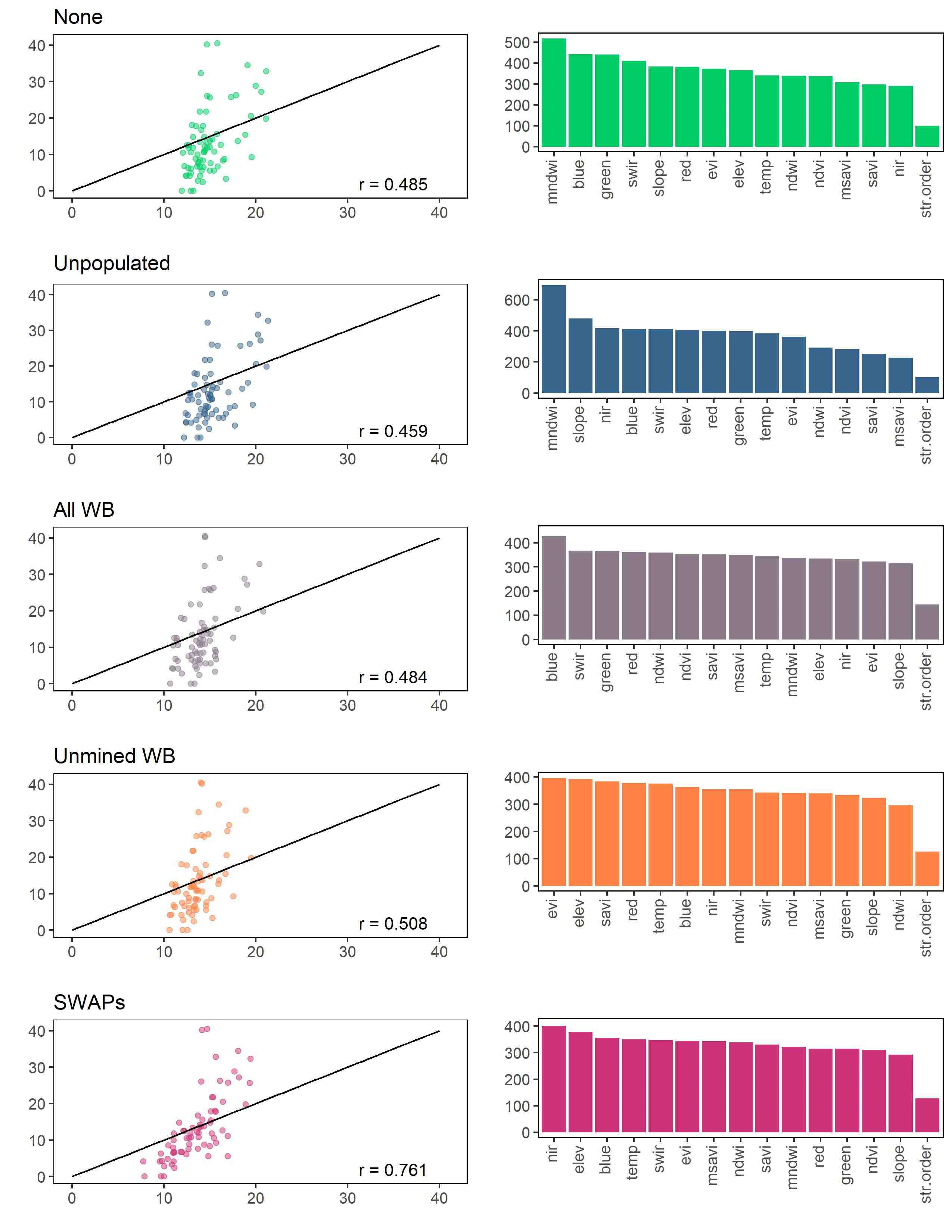

Supplement: S22 Fig — (TIF) [file pntd.0006517.s024.tif]

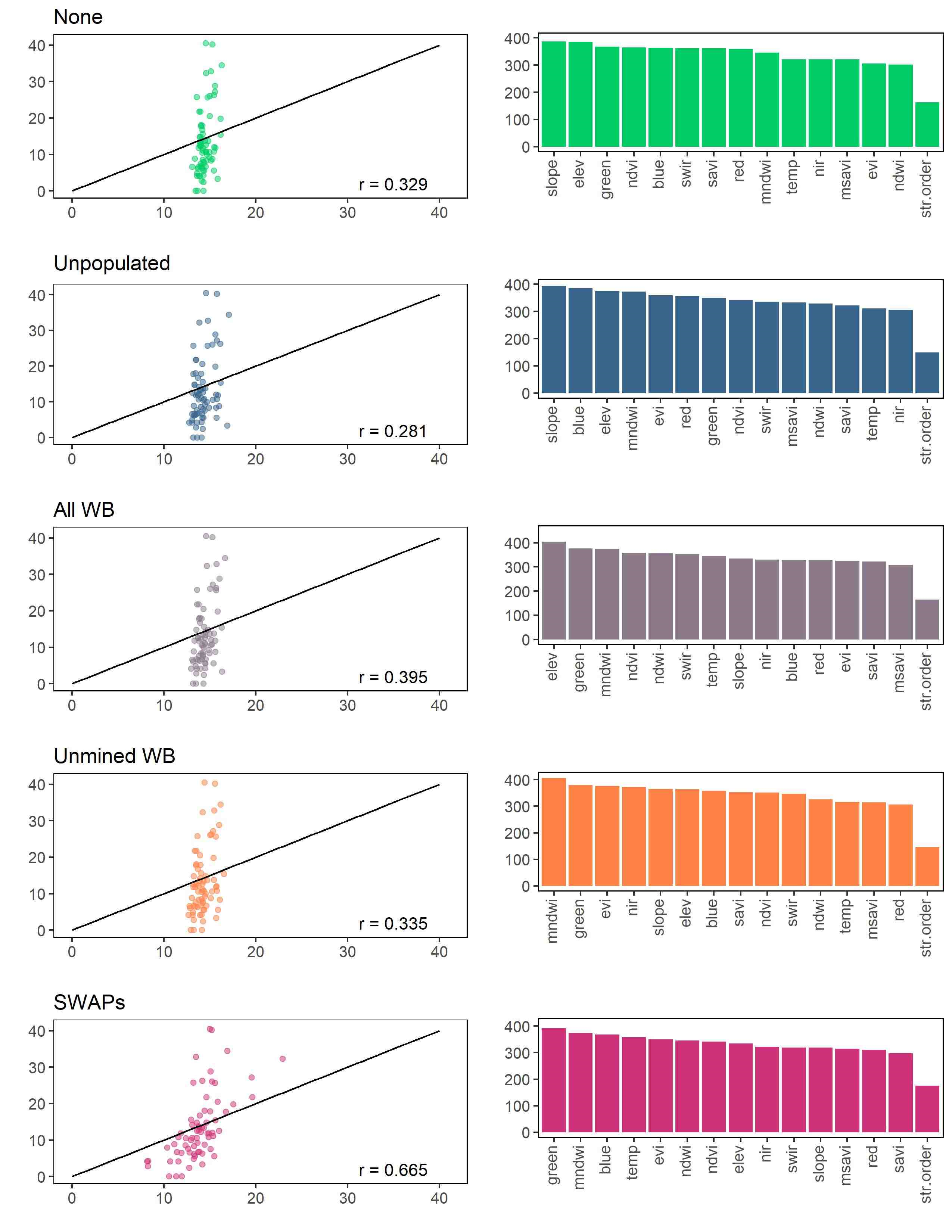

Supplement: S23 Fig — (TIF) [file pntd.0006517.s025.tif]

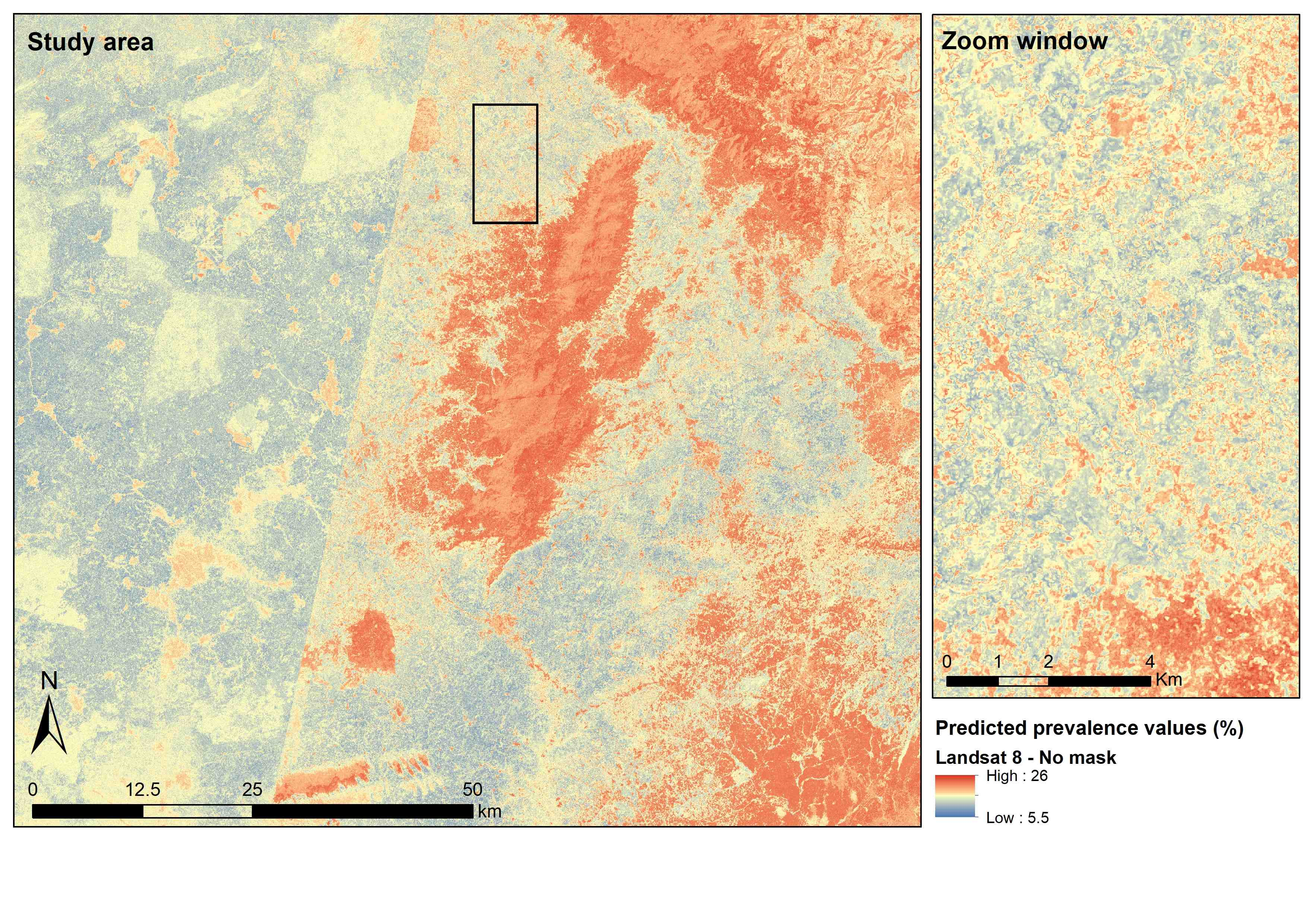

Supplement: S24 Fig — (TIF) [file pntd.0006517.s026.tif]

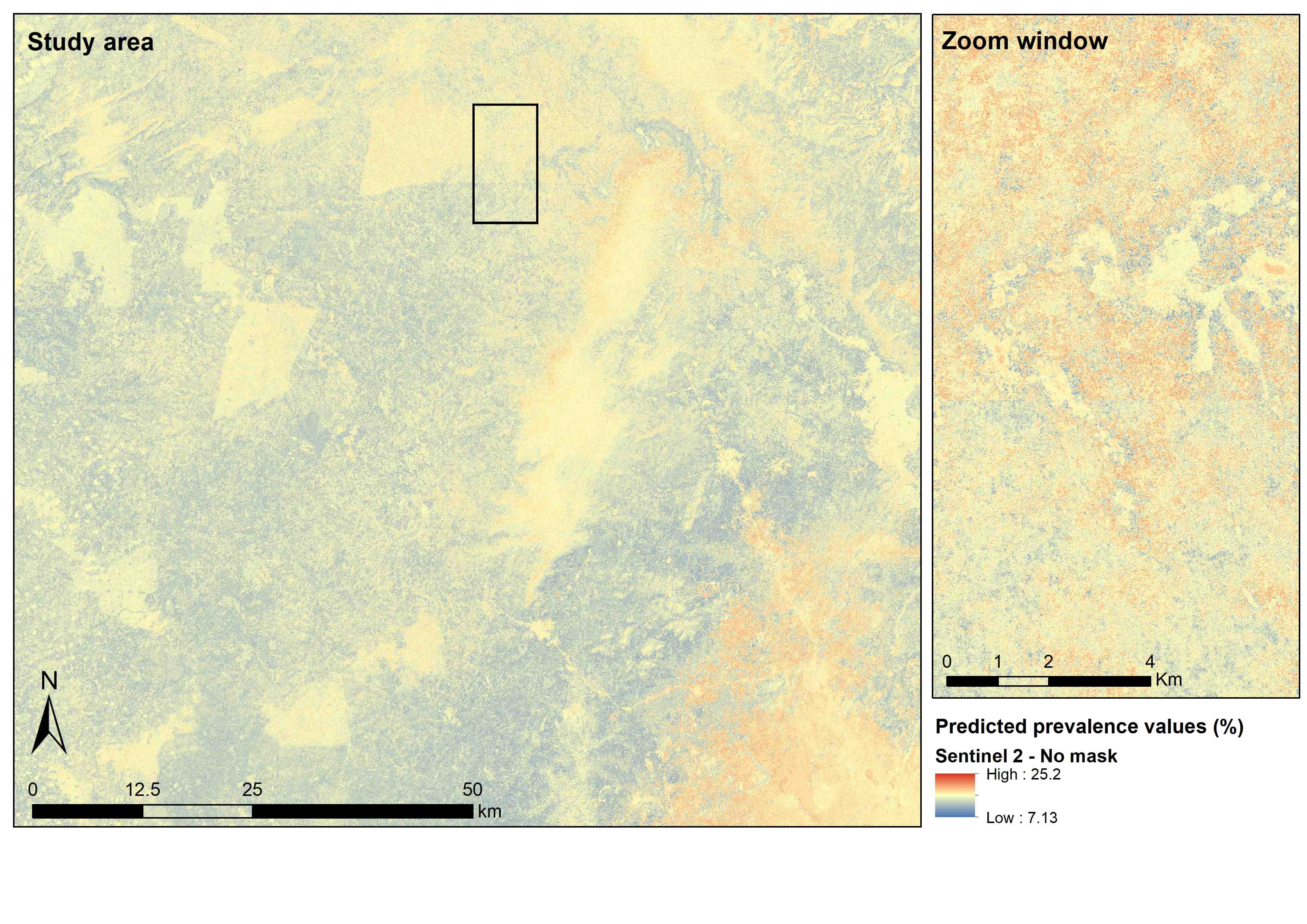

Supplement: S25 Fig — (TIF) [file pntd.0006517.s027.tif]

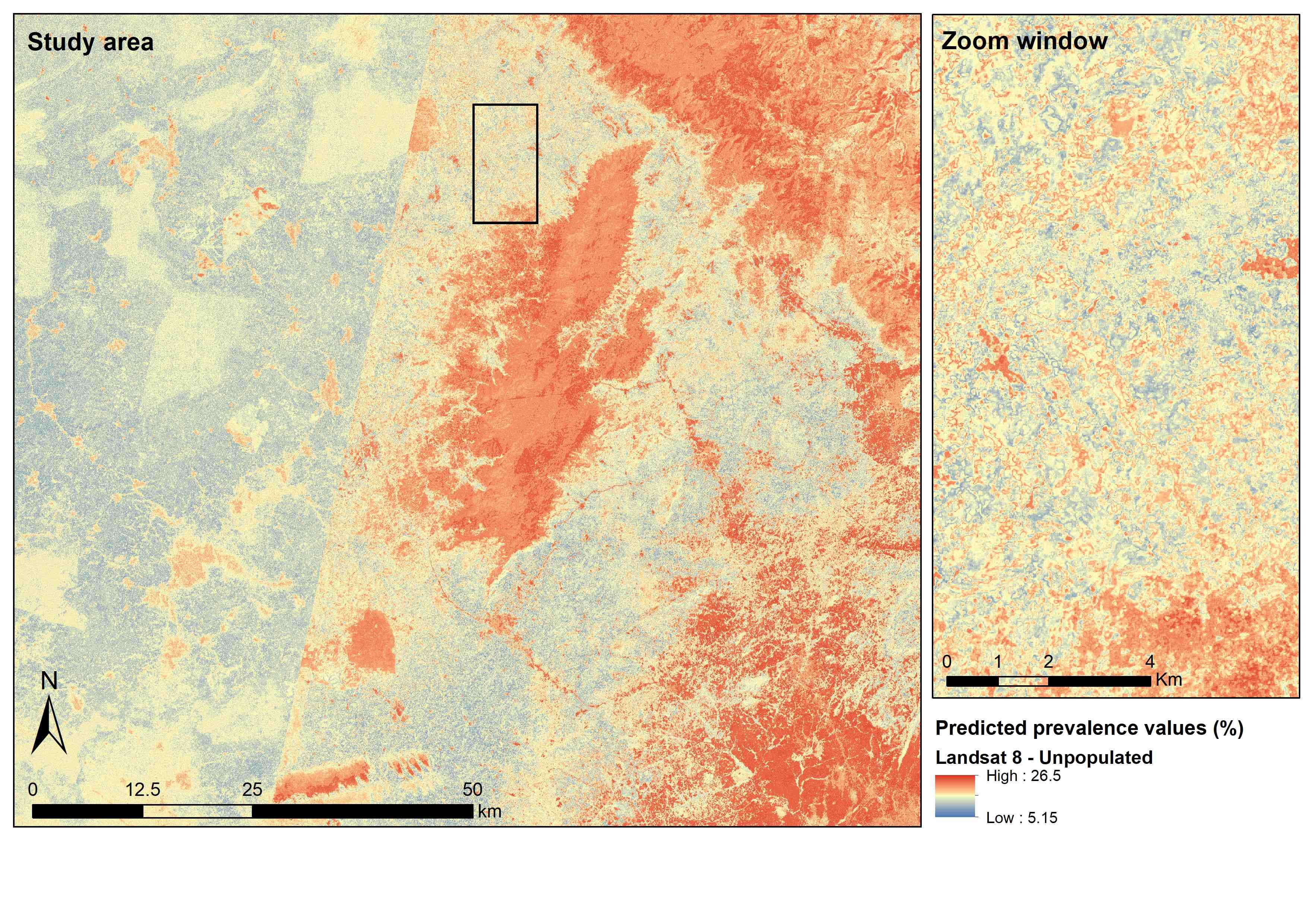

Supplement: S26 Fig — (TIF) [file pntd.0006517.s028.tif]

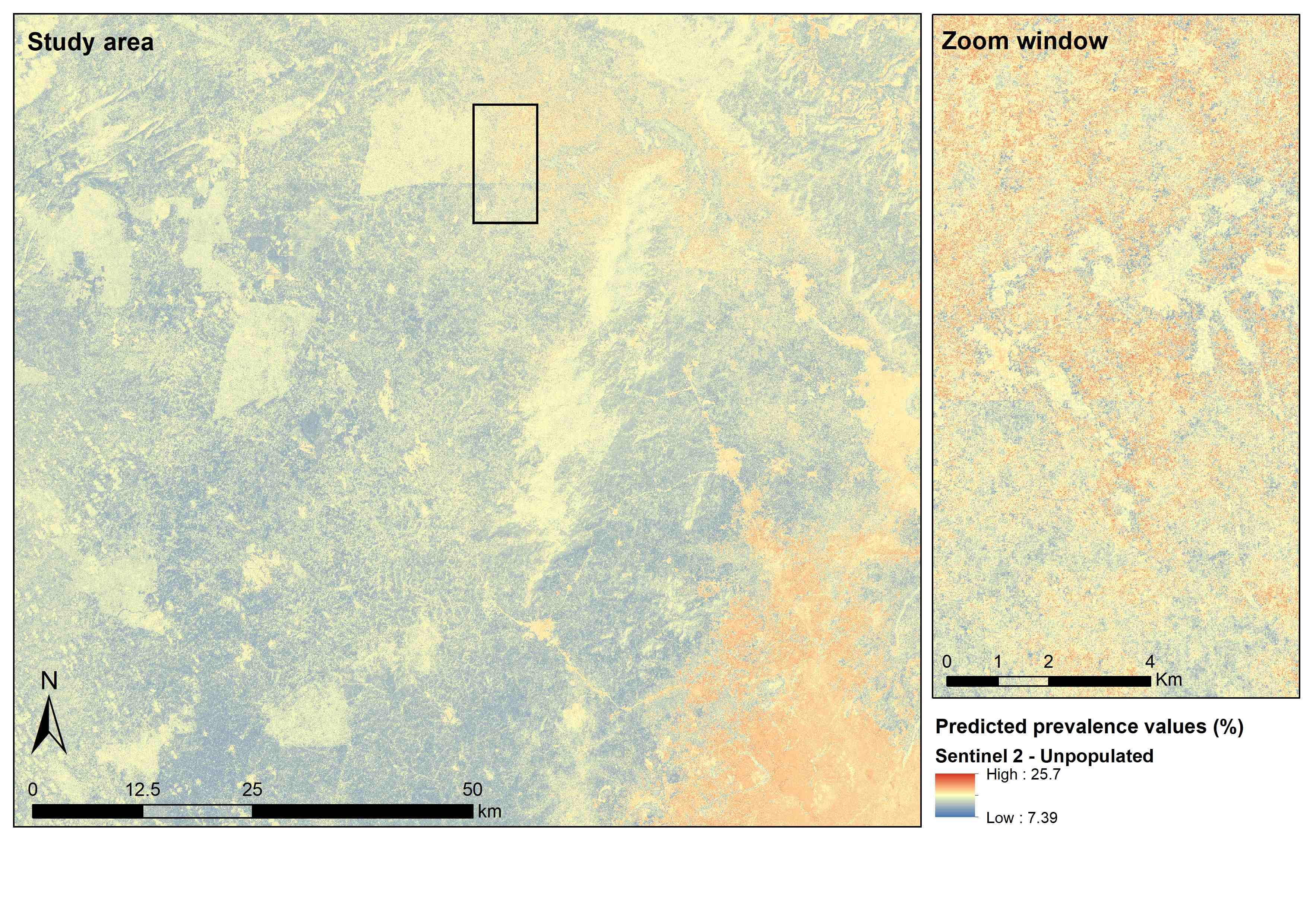

Supplement: S27 Fig — (TIF) [file pntd.0006517.s029.tif]

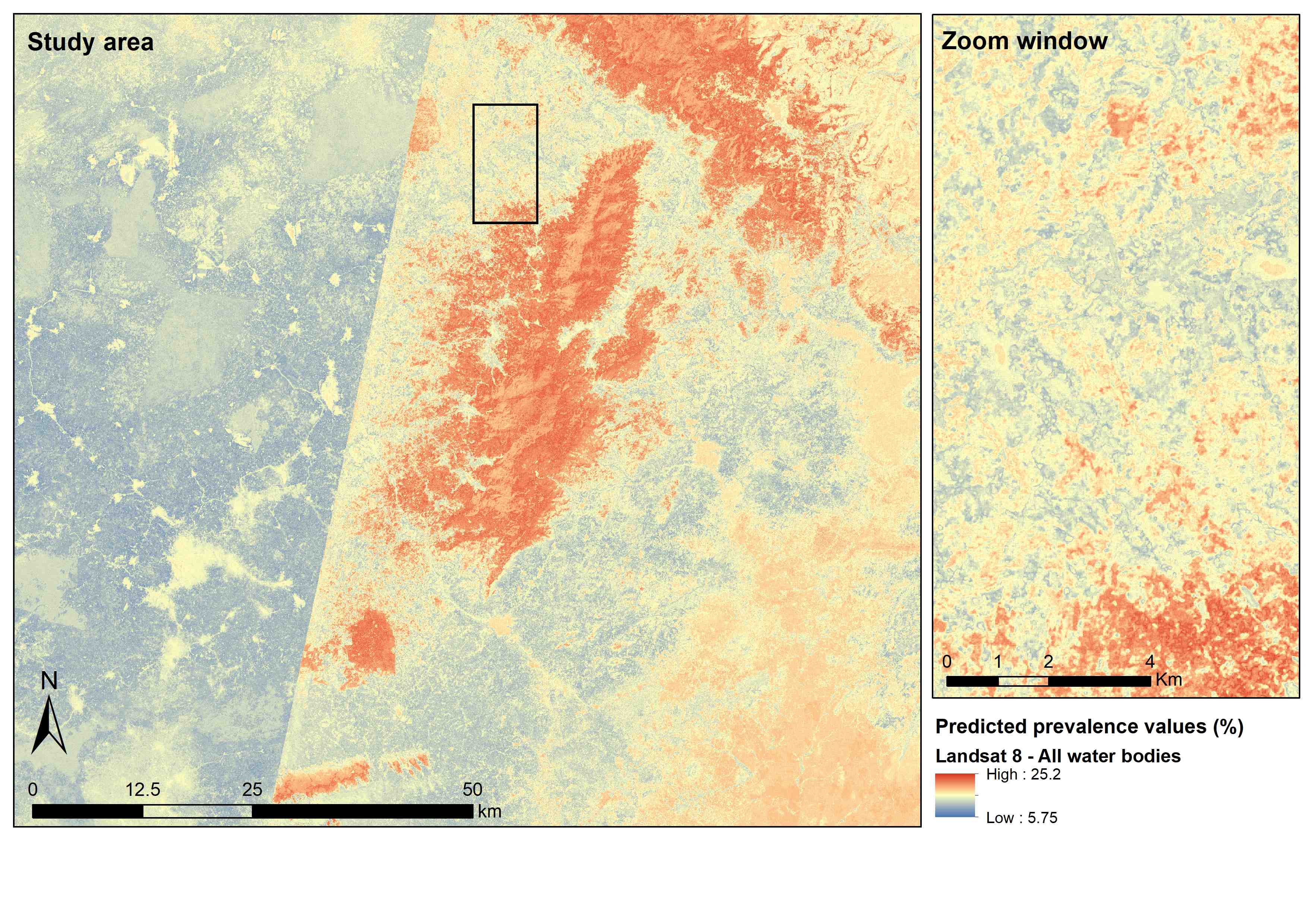

Supplement: S28 Fig — (TIF) [file pntd.0006517.s030.tif]

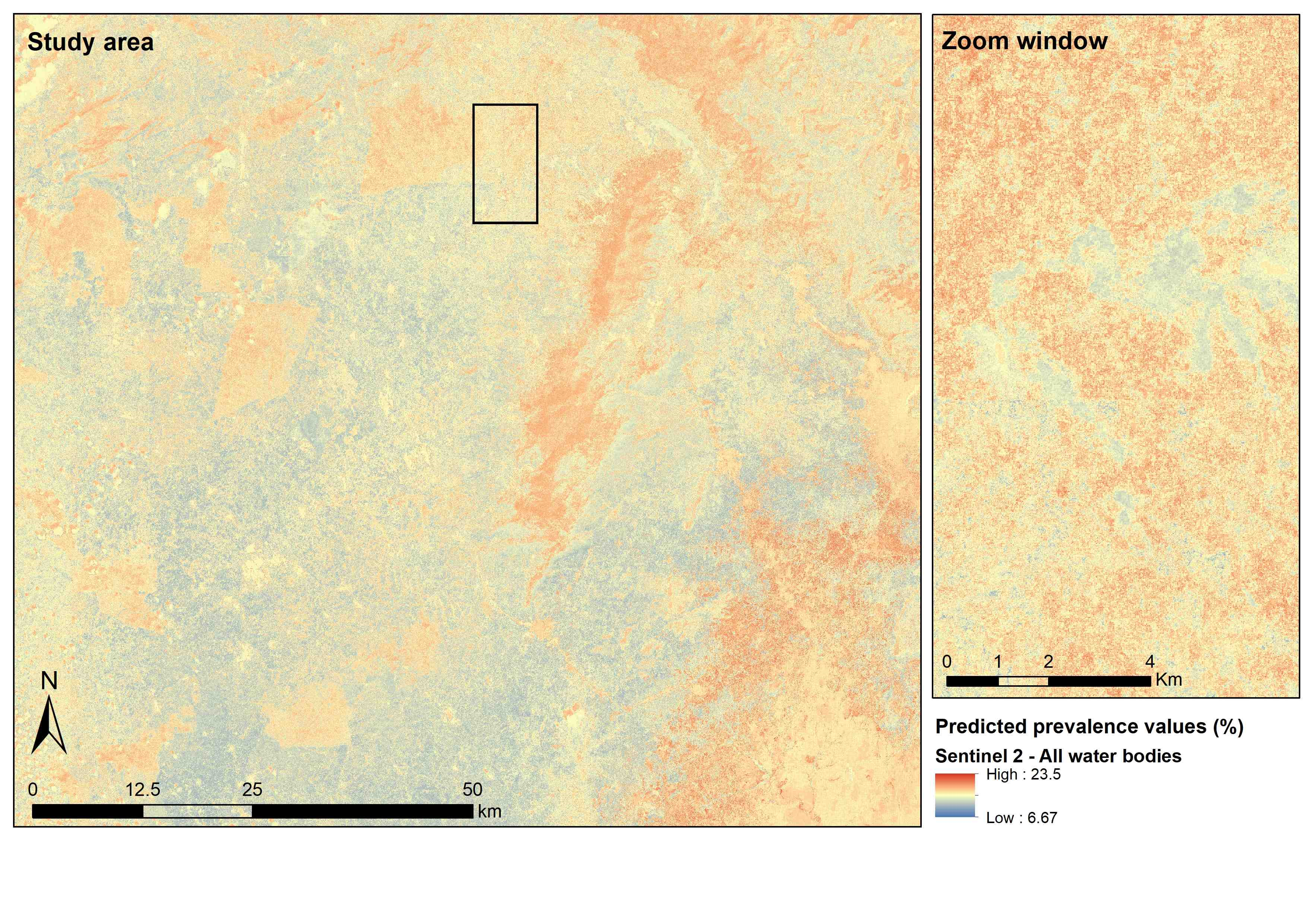

Supplement: S29 Fig — (TIF) [file pntd.0006517.s031.tif]

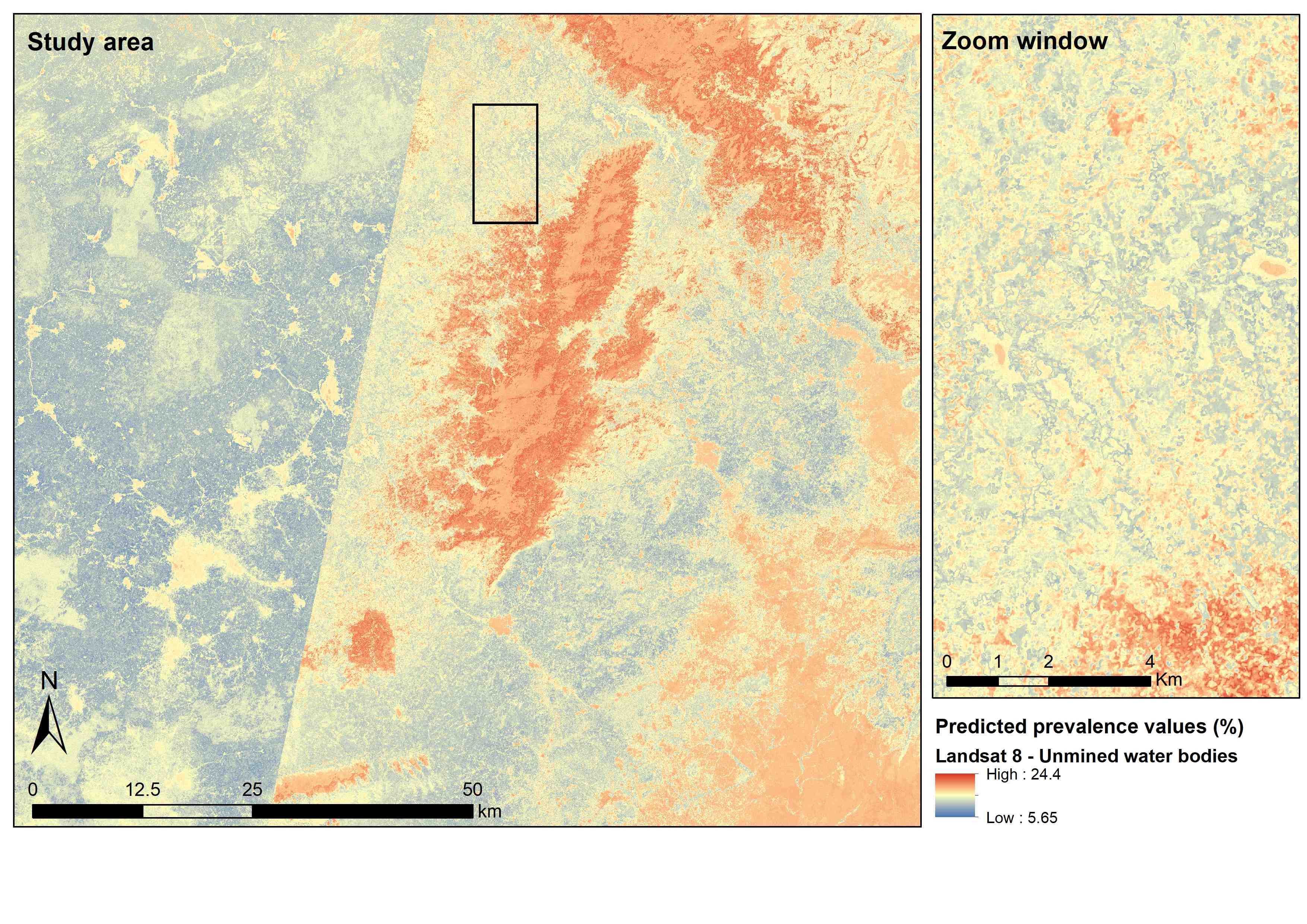

Supplement: S30 Fig — (TIF) [file pntd.0006517.s032.tif]

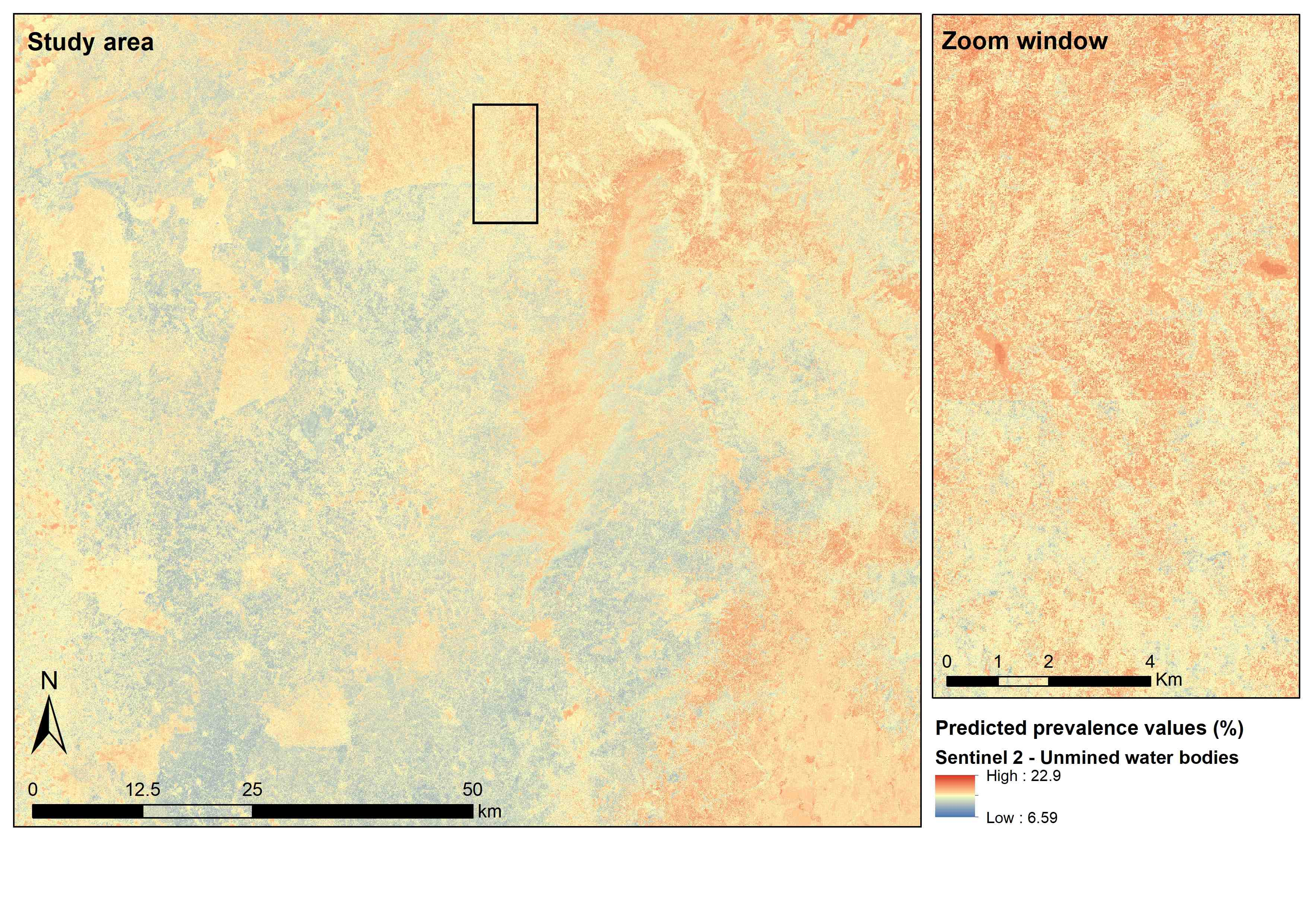

Supplement: S31 Fig — (TIF) [file pntd.0006517.s033.tif]

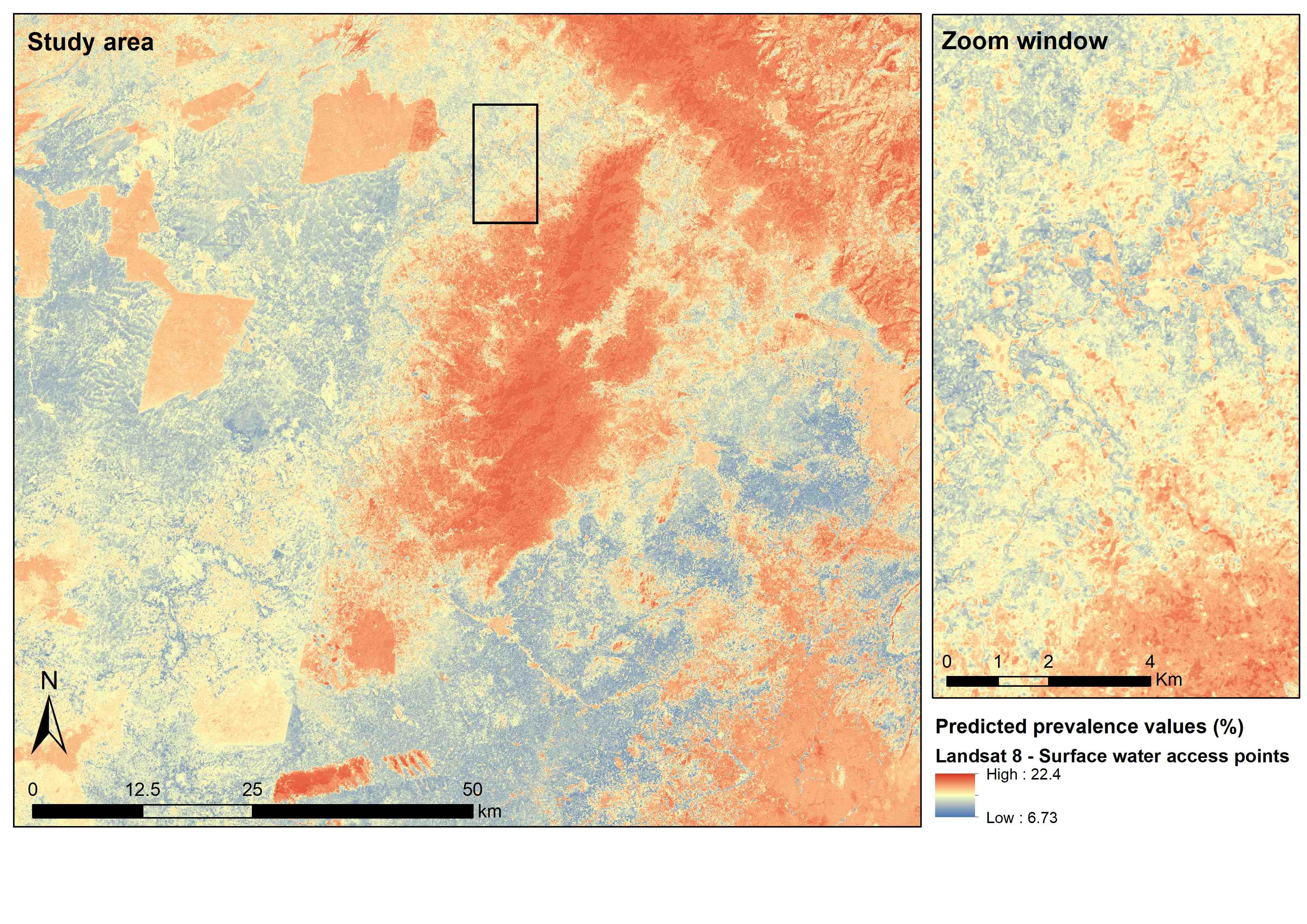

Supplement: S32 Fig — (TIF) [file pntd.0006517.s034.tif]

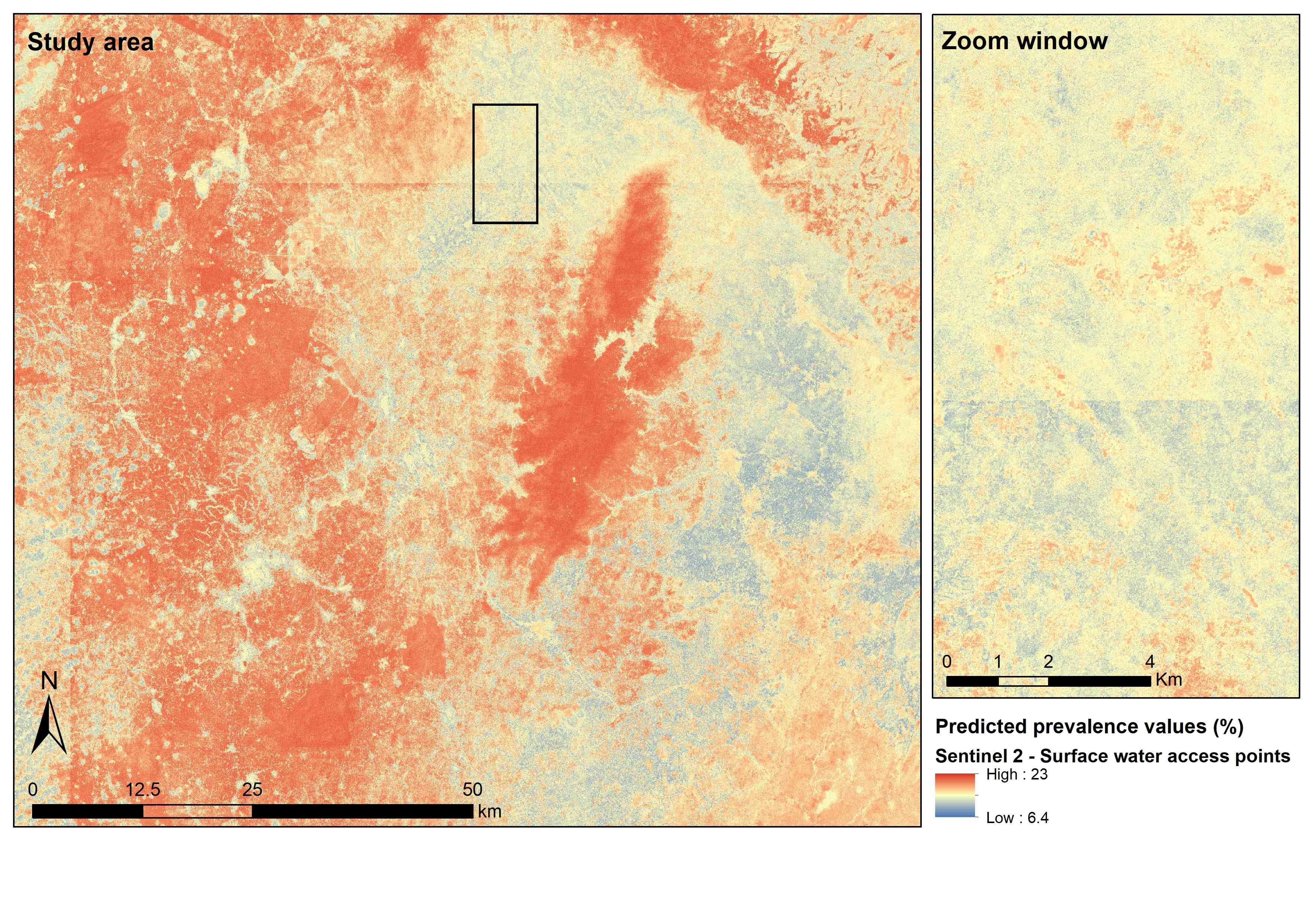

Supplement: S33 Fig — (TIF) [file pntd.0006517.s035.tif]
